# Adenine base editor correction of pathogenic variations associated with inherited retinal dystrophy in patient iPSC and retinal organoids

**DOI:** 10.1016/j.omtn.2025.102777

**Published:** 2025-11-17

**Authors:** Amy Leung, Pedro R.L. Perdigão, Almudena Sacristan-Reviriego, Paul E. Sladen, Erika A. Aguzzi, Farah.O. Rezek, Kalliopi Ziaka, Rosellina Guarascio, Kwan-Leong Hau, Michael E. Cheetham, Jacqueline van der Spuy

**Affiliations:** 1 University College London Institute of Ophthalmology, University College London, London EC1V 9EL, UK; 2 Centre for Neuroscience and Cell Biology, University of Coimbra, 3004-504 Coimbra, Portugal; 3 Center for Innovative Biomedicine and Biotechnology, University of Coimbra, 3004-504 Coimbra, Portugal; 4 Gene Therapy Center of Excellence Portugal, University of Coimbra, 3004-504 Coimbra, Portugal; 5 Biochemistry and Molecular Biology Department, Faculty of Medicine, Universidad Complutense de Madrid, 28040 Madrid, Spain

**Keywords:** MT: RNA/DNA editing, inherited retinal dystrophy, CRISPR, Cas9, base editing, retinal organoid, induced pluripotent stem cell, Leber congenital amaurosis

## Abstract

Inherited retinal dystrophies (IRDs) are a group of incurable, genetically heterogeneous diseases that cause progressive degeneration of the retina, leading to the loss of vision. Genome editing technologies offer a powerful prospect for mutation correction and single-dose cures for these diseases. Here, we investigated the potential of adenine base editing (ABE) to correct a panel of causative genetic variations in patient-derived induced pluripotent stem cells (iPSCs) and identified parameters that can efficiently correct a pathogenic variation in the *AIPL1* gene (c.665G>A, p.Trp222∗), which is associated with autosomal recessive Leber congenital amaurosis type 4. To investigate correction of the variant in a patient-relevant model, retinal organoids (ROs) were derived from corrected isogenic and patient-derived iPSCs. Adenine base editor components were delivered to ROs via lipofection as chemically modified RNA or via a split intein system following dual-AAV transduction. The data show AIPL1 rescue in photoreceptor cells with both delivery systems and restoration of the AIPL1 target protein, cyclic guanosine monophosphate phosphodiesterase 6—a critical component of the visual transduction system—in treated rod photoreceptors. These proof-of-principle experiments highlight the utility of ROs for investigating the potential of ABE technology as a means to treat IRDs.

## Graphical abstract

**Figure:**
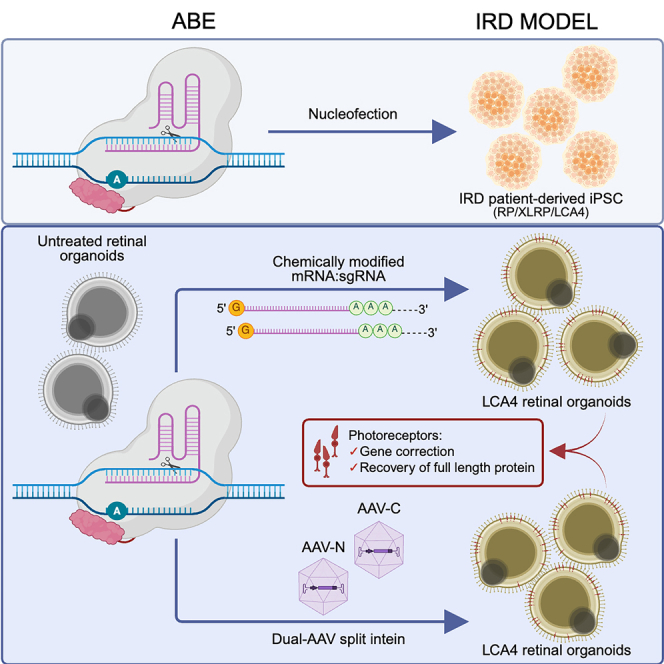

## Teaser

In this study, Leung and colleagues investigate precise gene correction by adenine base editing in patient-derived iPSCs and retinal organoids modeling inherited retinal dystrophy. The authors report effective gene correction in retinal organoids modeling Leber congenital amaurosis subtype 4 following dual-AAV split-intein or RNA delivery of the adenine base editor.

## Introduction

Base and prime editing are two recent advancements of clustered regularly interspaced short palindromic repeats (CRISPR)-CRISPR-associated protein 9 (Cas9) genome editing technologies, which hold great promise as methods to precisely correct disease-causing genetic variants.^1^^,^^2^ These technologies require significant optimization for clinical application; therefore, in this study, we investigated the utility of adenine base editing (ABE) to correct genetic variants associated with inherited retinal disease using human retinal organoid (RO) models.

Adenine base editors (ABEs), which comprise an adenine deaminase domain fused to Cas9 nickase, mediate the conversion of adenine to guanine in a precise, targeted manner at a site of interest in genomic DNA, as delineated by the 20-nt protospacer sequence of the ABE single-guide RNA (sgRNA), without the need for an exogenous repair template.^1^ Because the editing system does not involve the creation of double-strand breaks (DSBs) in genomic DNA, the risk of deleterious insertions and deletions caused by nonhomologous end-joining (NHEJ) or larger-scale changes, such as chromosomal translocations, is greatly reduced compared to Cas9 nuclease activity.^3^^,^^4^ Unlike CRISPR-Cas9 homology-directed repair (HDR), which requires an exogenous repair template and occurs primarily during the S and G2 phases of the cell cycle—rendering it highly inefficient in nondividing cells—base editing can occur in nondividing, mature cells. Altogether, these characteristics of ABEs render them a technology with great clinical potential for the targeted correction of deleterious mutations in patient tissues. Indeed, approximately 50% of identified pathogenic single-nucleotide variants in the human genome involve a G>A conversion (ClinVar: https://www.ncbi.nlm.nih.gov/clinvar/, accessed February 4, 2025), and ABE technology therefore holds great promise as a method to precisely correct these disease-causing variants.^1^ Since the description of the first base editors, a multitude of ABE variants have been engineered, with marked improvements in editing efficiencies,^5^^,^^6^^,^^7^ alterations to protospacer adjacent motif (PAM) site preferences,^8^^,^^9^ and modified editing window sizes.^7^^,^^8^^,^^10^ The ability of base editing to correct deleterious genetic variants has been tested in a wide range of *in vivo* models for heritable diseases, including inherited retinal dystrophies (IRDs).^11^^,^^12^^,^^13^^,^^14^^,^^15^^,^^16^^,^^17^^,^^18^^,^^19^^,^^20^^,^^21^^,^^22^

IRDs comprise a wide variety of genetic disorders with variable clinical outcomes, involving over 320 retinal disease genes primarily associated with photoreceptor and retinal pigment epithelium (RPE) function (RetNet: https://RetNet.org/, accessed February 4, 2025). IRDs often lead to severe visual impairment or blindness in childhood, adolescence, or early adulthood, affecting an estimated 5.5 million people worldwide and representing a significant burden on individuals and society.^23^ While many interventional clinical trials for IRDs have been completed or are ongoing, only one gene therapy product, voretigene neparvovec-rzyl (Luxturna), has been approved for the treatment of patients with biallelic genetic variations in the *RPE65* gene, typically diagnosed as Leber congenital amaurosis (LCA) type 2 or severe early-onset retinitis pigmentosa (RP).^24^^,^^25^ Therefore, there is a strong unmet need for novel therapeutics for the overwhelming majority of patients with genetically heterogeneous IRDs. Approaches that require minimum treatments or surgeries, such as gene correction, are ideal given the difficulty of therapeutic administration to the posterior eye.

In this study, we examined a panel of patient IRD-associated genetic variations to identify potential targets for correction by ABE. After initial testing *in vitro* in patient-derived induced pluripotent stem cell (iPSC) lines, we observed highly efficient base editing of *AIPL1* c.665G>A, p.Trp222∗, a nonsense mutation associated with LCA type 4 (LCA4). To test the delivery of base editing components to photoreceptors, mRNA/sgRNA lipofection and dual-AAV delivery were characterized in RO models from LCA4 patient iPSCs, with both systems able to restore AIPL1 protein expression in edited photoreceptors. Additionally, some downstream effects of AIPL1 restoration were also apparent in the RO model. Overall, our study illustrates that while ABE is a viable option for gene correction in IRDs, the use of human ROs modeling IRDs highlights the need to overcome challenges in specificity, efficiency, and delivery required for clinical translation.

## Results

### Correction of LCA4 *AIPL1* variant through ABE in patient iPSCs

**Figure 1:**
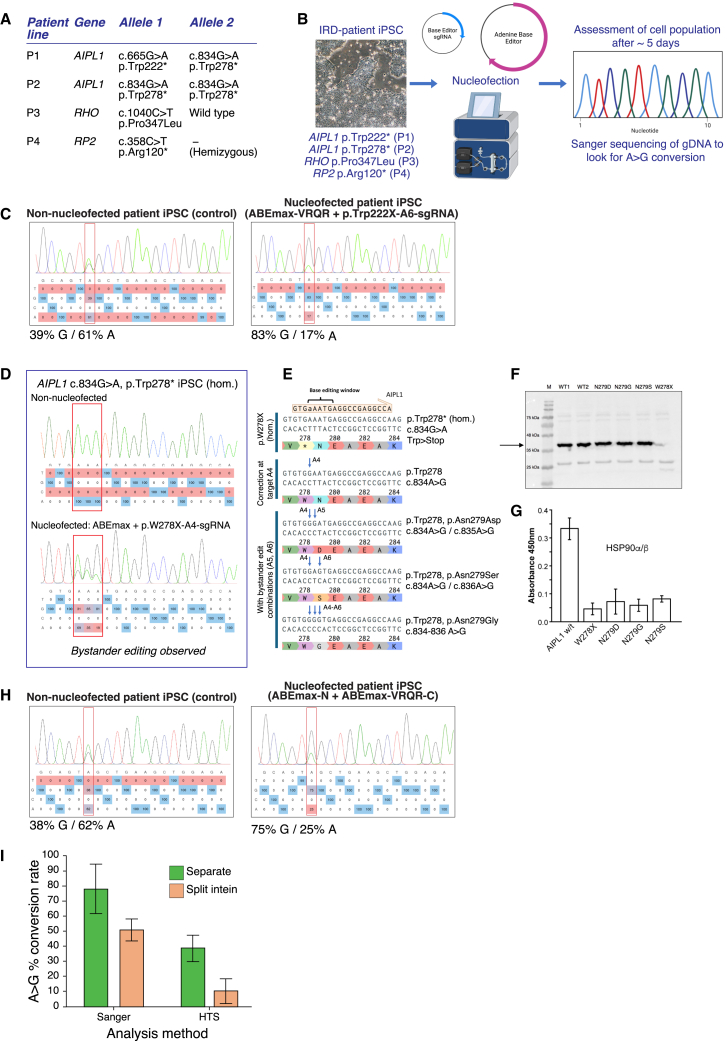
Adenine base editing can correct the LCA4 genetic variant *AIPL1* c.665G>A,p.Trp222∗ (A) Genotype of patient iPSC lines. (B) Screening of ABEs and sgRNAs in IRD patient iPSC lines. iPSCs were nucleofected with combinations of base editor and sgRNA expression plasmids. Approximately 5 days after nucleofection, iPSCs were screened by PCR and Sanger sequencing to assess whether base editing had occurred. (C) Adenine base editing of the *AIPL1* c.665G>A, p.Trp222∗ locus in p.Trp222∗/p.Trp278∗ compound heterozygous LCA4 patient iPSC cells. Sanger sequencing traces and EditR analysis of the *AIPL1* c.665G>A, p.Trp222∗ region show a shift in the proportion of A to G at position A6 (corresponding to position c.665) in ABE-expression plasmid (*AIPL1* c.6665G>A-A6-sgRNA + ABEmax-VRQR) nucleofected cells compared to non-nucleofected cells. Representative sequencing trace from *n* = three repeats. (D) Significant bystander editing at the AIPL1 c.834G>A, p.Trp278∗ locus with ABEmax and AIPL1 c.834G>A-A4-sgRNA. Analysis of gDNA from patient iPSC (c.834G>A, p.Trp278∗ [hom]) nucleofected with ABE component expression plasmids showed editing at the target adenine (A4) and also significant, more efficient editing of A>G at the bystander positions A5 and A6. No base editing was observed for other AIPL1 c.834G>A, p.Trp278∗ sgRNAs and ABE combinations tested (data not shown). (E) Bystander editing of A>G at positions A5 and A6 within the AIPL1 c.834G>A, p.Trp278∗ locus is predicted to lead to deleterious changes to AIPL1 protein at amino acid position 279 (Asn279>Asp/Ser/Gly). (F) Bystander editing at the AIPL1 c.834G>A, p.Trp278∗ locus leads to the expression of full-length AIPL1 p.Asn279Asp (p.N279D), p.Asn279Ser (p.N279S), and p.Asn279Gly (p.N279G) (∼43kDa). AIPL1 p.Trp278∗ forms insoluble aggregates that are not detected by western blotting (data not shown). (G) AIPL1 p.Asn279Asp (p.N279D), p.Asn279Ser (p.N279S), and p.Asn279Gly (p.N279G) proteins are unable to interact with HSP90 and are therefore functionally deficient. Graph shows mean ± SD. (H) Sanger sequencing traces and EditR analysis of the *AIPL1* c.665G>A, p.Trp222∗ region show a shift in the proportion of A to G at position A6 (corresponding to position c.665) in split intein-expression plasmid (ABEmax-N + ABEmax-VRQR-C) nucleofected LCA4 patient iPSCs compared to non-nucleofected cells. Representative sequencing trace from *n* = three repeats. (I) Graphical representation of the differences in editing efficiencies (conversion rates of A to G), as estimated by Sanger sequencing followed by EditR analysis or by HTS (data calculated from A/G values from *n* = three independent experiments, compared with non-nucleofected control samples). Graph shows mean ± SD. Sanger sequencing overrepresents the degree of editing in samples following both nucleofection of separate ABE expression plasmids and split intein expression plasmids.

A selection of IRD patient-derived iPSC lines were screened for their potential suitability for ABE gene correction (Figure 1A).^26^^,^^27^ Variations in *AIPL1* (p.Trp222∗; c.834G>A, p.Trp278∗), *RHO* (c.1040C>T, p.Pro347Leu), and *RP2* (c.358C>T, p.Arg120∗) were identified, all caused by a G>A transition on the sense or complementary strand of the gene (Figures 1A and S1A; Table S1). The loss-of-function *AIPL1* variations c.665G>A, p.Trp222∗ and c.834G>A, p.Trp278∗ are associated with LCA4, a rare early-onset autosomal recessive disorder leading to vision loss within the first few years of life.^28^ *RHO* c.1040C>T, p.Pro347Leu is a common variant in the UK population causing autosomal dominant RP, and *RP2* c.358C>T, p.Arg120∗ is a common stop mutation and mutational hotspot causing X-linked RP—both of which cannot be treated with existing gene therapy approaches.^29^^,^^30^ The IRD variations selected all met the criteria for optimal editing, including an optimally located PAM that positioned the target variation at the optimal location (positions 5 or 6) within the editing window of the ABEmax SpCas9 family of base editors (positions A4 to A7 of the guide RNA)^5^ and, with the exception of *AIPL1* c.834G>A, p.Trp278∗, the absence of runs of A or T within the editing window to avoid bystander editing of adjacent bases (Figure S1A). Positions of ABE-sgRNAs were mapped for each genetic variant to match the PAM requirement, ensuring that the target adenine fell within the A4–A7 editing window of the sgRNA protospacer (Figure S1A; Table S2). Accordingly, ABEmax (NGG PAM) or variants thereof—ABEmax-NG (NG PAM), ABEmax-VRQR (NGA PAM), and ABEmax-SpRY (near PAMless)—were paired with the sgRNA.^8^^,^^9^ To test the ABE efficiency of sgRNA and base editor combinations, IRD patient iPSCs were nucleofected with separate expression plasmids for these two components (Figure 1B). The resultant iPSC cultures were screened at the relevant loci through Sanger sequencing of the gDNA amplicon from the mixed cell population. In total, out of the nine sgRNAs designed for the four IRD genetic variants (three sgRNAs for *AIPL1* c.834G>A, p.Trp278∗, one for *AIPL1* c.665G>A, p.Trp222∗, one for *RHO* c.1040C>T, p.Pro347Leu, and four for *RP2* c.358C>T, p.Arg120∗) (Figure S1A), clear signs of editing were observed for only two sgRNAs, both targeting *AIPL1* (*AIPL1* c.665G>A-A6-sgRNA and *AIPL1* c.834G>A-A4-sgRNA).

For *AIPL1* c.665G>A, p.Trp222∗, the c.665G>A-A6-sgRNA and ABEmax-VRQR combination was tested in patient iPSCs compound heterozygous for c.665G>A, p.Trp222∗ and c.834G>A, and p.Trp278∗ (Figures 1A and S1A).^27^ A shift in the proportion of A to G at the c.665G>A, p.Trp222∗ site was observed in nucleofected iPSCs (Figure 1C), indicating that base editing had occurred. Calculations from EditR analyses^31^ of peak height estimated the conversion rate of A>G at 78.2% ± 16.3%, based on three individual samples (83%G/17%A; 79%G/21%A; 98%G/2%A), compared to non-nucleofected patient samples (38%G/62%A; 39%G/61%A; 38%G/62%A). No bystander editing was detected by Sanger sequencing.

For *AIPL1* c.834G>A, p.Trp278∗, the *AIPL1* c.834G>A-A4-sgRNA in combination with ABEmax resulted in clear A>G conversion at c.834, with an estimated 31% of A converted to G at this locus (Figure 1D). However, more efficient base editing was observed at the adjacent adenines at positions A5 (65%) and A6 (81%) (Figure 1D). These bystander edits are predicted to result in deleterious changes to the AIPL1 coding sequence at amino acid (aa) 279, resulting in Asn279Asp, Asn279Ser, or Asn279Gly (Figure 1E). Full-length AIPL1 Asn279Asp, Asn279Ser, and Asn279Gly variants were shown to be stable following expression in HEK293T cells (Figure 1F) but were unable to bind the AIPL1 binding partner HSP90 (Figure 1G), confirming that bystander editing was functionally deleterious. sgRNAs designed to work in conjunction with ABEmax-SpRY did not result in observable base editing (data not shown).

For *RHO* c.1040C>T, p.Pro347Leu and *RP2* c.358C>T, p.Arg120∗, no editing was observed with any of the sgRNAs and ABE combinations tested (Figures S1B and S1C), despite pairing *RP2* c.358C>T-A4-sgRNA and c.358C>T-A5-sgRNA with the “near-PAMless” ABEmax-SpRY. Out of the four IRD genetic variants tested, *AIPL1* c.665G>A, p.Trp222∗ was therefore the most promising candidate for correction by ABE due to the high efficiency of editing at the target adenine and the absence of obvious deleterious bystander editing.

### Correction of LCA4 *AIPL1* variant through split-intein ABE in patient iPSCs

Having shown that ABEmax-VRQR and *AIPL1* c.665G>A-A6-sgRNA can achieve robust base editing of *AIPL1* c.665G>A, p.Trp222∗ in patient iPSCs, we designed and engineered CMV-driven constructs, adapted from Villiger et al., encoding these components within a split system using *Npu* DnaE inteins (Figure S2A).^32^ This strategy allows reconstitution of the large ABE framework via protein *trans*-splicing following AAV delivery. The ABEmax-VRQR coding sequence was split into an N-terminal half (2961bp/987 aa) and a C-terminal half (2526bp/842 aa), named ABEmax-N and ABEmax-VRQR-C, respectively. ABEmax-N was fused to a C-terminal DnaE N-intein, while ABEmax-VRQR-C was fused to an N-terminal DnaE C-intein. A U6-driven *AIPL1* c.665G>A-A6-sgRNA cassette was inserted downstream of the ABEmax-VRQR-C coding sequence (Figure S2A). Validation of the split-intein system was carried out through transfection of expression plasmids in HEK293T cells. ABEmax-N and ABEmaxVRQR-C could be detected using antibodies against Cas9 (raised against the N terminus of SpCas9) and FLAG, respectively (Figures S2B and S2C), with some instability of ABEmax-VRQR-C noted by western blotting. However, the recombined full-length ABEmax-VRQR was detectable by western blot analysis of protein extracts from HEK293T cells transfected with both expression plasmids (Figure S2C).

Nucleofection of LCA4 patient iPSCs, compound heterozygous for *AIPL1* c.665G>A, p.Trp222∗ and c.834G>A, p.Trp278∗, with split-intein pCMV-ABEmax-N and pCMV-ABEmaxVRQR-C expression plasmids also resulted in a shift in the proportion of A>G at *AIPL1* c.665 (Figure 1H), although it appeared to be less efficient compared to the results seen with full-length ABEmax-VRQR and sgRNA expression plasmids. EditR analysis of sequencing traces estimated the conversion rate of A>G at 51.0% ± 7.21% with the split-intein constructs—calculated from three individual samples (65%G/34%A; 69%G/31%A; and 75%G/25%A)—compared to the readings for non-nucleofected control samples.

To quantitatively determine editing efficiency, high-throughput amplicon sequencing (HTS) was carried out on patient iPSC samples nucleofected with either full-length expression plasmids or split-intein plasmids for ABEmax-VRQR and *AIPL1* c.665G>A-A6-sgRNA. The rate of A>G conversion calculated from HTS was lower than that estimated by Sanger sequencing and EditR analysis (Figure 1I). Samples nucleofected with full-length expression plasmids displayed a conversion rate of 38.9% ± 8.73%, while split-intein samples displayed a comparatively lower conversion rate of 10.61% ± 8.11% (Figure 1I). In addition, HTS revealed low-level editing outside of the A4–A7 window expected for ABEmax-VRQR, with some bystander editing observed at position A3 (Figure S2D). A residual percentage of amplicon products with editing at both bystander A3 and target A6 was detected for both separate expression plasmids and split-intein plasmid nucleofected samples by HTS; 0.39% ± 0.09% and 0.11% ± 0.08% of total amplicons, respectively (Figure S2D). The resultant nonsynonymous change at A3 (c.662A>G, p.Gln221Arg) is potentially deleterious, with a CADD (PHRED) score of 17.80.

Finally, Cas-OFFinder (Cas-OFFinder: http://www.rgenome.net/cas-offinder/)^33^ was used to identify potential off-target editing sites within the human genome for the combination of *AIPL1* c.665G>A-A6-sgRNA and ABEmax-VRQR. The top 10 candidate genes (with the fewest number of mismatches and smallest bulge sizes) were selected for further screening by Sanger sequencing and HTS (Table S3). While no changes were detected by Sanger sequencing (data not shown), a small degree of off-target base editing was detected at off-target site 9 (OT9) by HTS (0.26% of reads with separate plasmids) (Figure S3). OT9 is not located within a gene coding sequence, and the small degree of base editing detected is therefore unlikely to affect gene function or cell activity.

### Generation of *AIPL1* isogenic iPSCs

**Figure 2:**
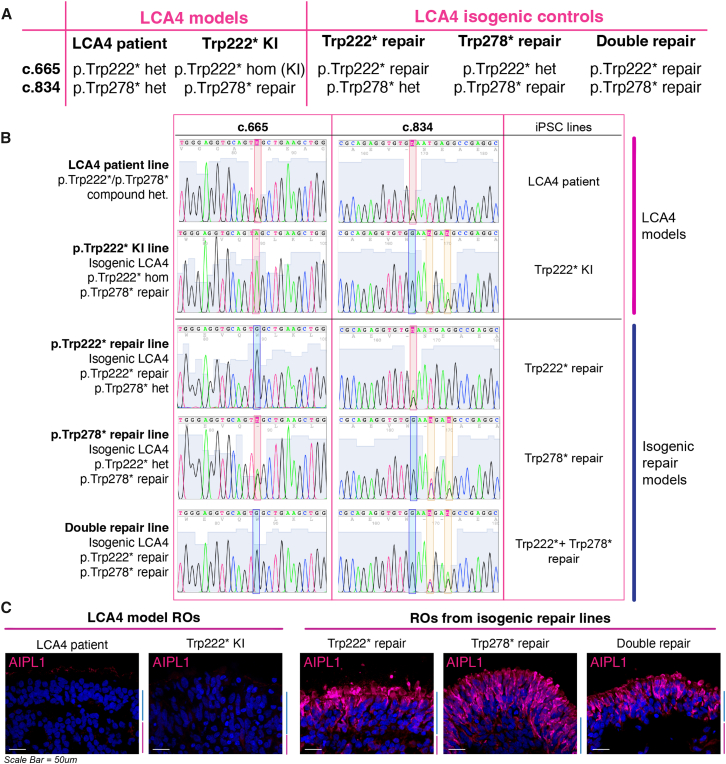
Generation of LCA4 isogenic iPSC lines and characterization of AIPL1 expression in retinal organoids (A) LCA4 isogenic lines were generated by gene editing at the AIPL1 p.Trp222∗ and p.Trp278∗ loci. Gene editing at these loci was used to create four isogenic lines from LCA4 patient iPSCs, including three isogenic repair lines and an alternate LCA4 model: Trp278∗ repair (p.Trp222∗ het), Trp222∗ repair (p.Trp278∗ het), double Trp222∗/Trp278∗ repair (repaired at both loci), and Trp222∗ knock in (KI) LCA4 model (homozygous for p.Trp222∗ with repair at p.Trp278∗). (B) Sanger sequencing chromatograms of the p.Trp222 and p.Trp278 loci in all LCA4 model and isogenic iPSC lines are shown. The Trp222∗ KI and compound heterozygous variants at the two loci are demarcated by red bars; repair at the two loci is demarcated by blue bars. Two synonymous changes in the PAM are introduced downstream of c.834 at the p.Trp278 locus (yellow bars). iPSC lines are referred to by the names in the far-right column: LCA4 patient and Trp222∗ KI (LCA4 models); Trp222∗ repair, Trp278∗ repair, and Trp222∗/Trp278∗ double repair (isogenic repair models). (C) AIPL1 ICC in D255 ROs derived from LCA4 model and LCA4 repair iPSCs. LCA4 model ROs lack AIPL1 immunogenicity in contrast to all LCA4 isogenic repair ROs, where expression is localized to the photoreceptor cell layer (the ONL). There was no discernible difference in AIPL1 signal between the double repair ROs and the heterozygous repair ROs. The ONL and inner nuclear layer (INL) regions of the RO sections are highlighted by blue and magenta lines, respectively, next to each image. DAPI in blue. Scale bars, 50 μm.

A comprehensive range of isogenic iPSC lines was generated from the p.Trp222∗/p.Trp278∗ compound heterozygous patient iPSC to provide genetically matched lines for *in vitro* modeling of LCA4 using the iPSC-RO model (Figure 2). The p.Trp222∗/p.Trp278∗ parental iPSC line was previously characterized for pluripotency and trilineage potential, and we have previously demonstrated effective isogenic repair of a homozygous *AIPL1* c.834G>A, p.Trp278∗ iPSC line through CRISPR-Cas9-mediated HDR.^27^ Repair of *AIPL1* c.665G>A, p.Trp222∗ in the compound heterozygous LCA patient iPSC line was achieved using ABE with ABEmax-VRQR and *AIPL1* c.665G>A-A6-sgRNA, while a CRISPR-Cas9-HDR strategy was used to knock in (KI) c.665G>A, p.Trp222∗ (Figure S4; Table S4). These three gene-editing approaches were employed to create three different isogenic repair iPSC lines from the compound heterozygous LCA4 patient line (Trp222∗ repair, Trp278∗ repair, and Trp222∗+Trp278∗ double repair) and iPSCs homozygous for p.Trp222∗ (Trp222∗ KI, Trp278∗ repair) (Figures 2A and 2B). Pluripotency was confirmed, and no off-target editing was detected by Sanger sequencing of the top 10 predicted sites (Cas-OFFinder) in any of the iPSC lines (data not shown).

AIPL1 expression is normally localized to the rod and cone photoreceptors in the outer nuclear layer (ONL) of iPSC-ROs and can be detected from approximately day (D) 98 of RO differentiation but is absent in LCA4 AIPL1 loss-of-function models.^27^^,^^34^^,^^35^^,^^36^ Characterization of AIPL1 immunoreactivity in ROs derived from LCA4 isogenic iPSC lines revealed strong AIPL1 staining localized to the ONL in all three types of isogenic repair ROs. As previously characterized, LCA4 ROs lack detectable AIPL1, and this was also the case for Trp222∗ KI ROs (Figure 2C). The isogenic lines created through gene editing of AIPL1 therefore display the expected patterns of AIPL1 expression in the RO model.

### Comparison of lipofection or AAV-mediated delivery of base editing components to ROs

**Figure 3:**
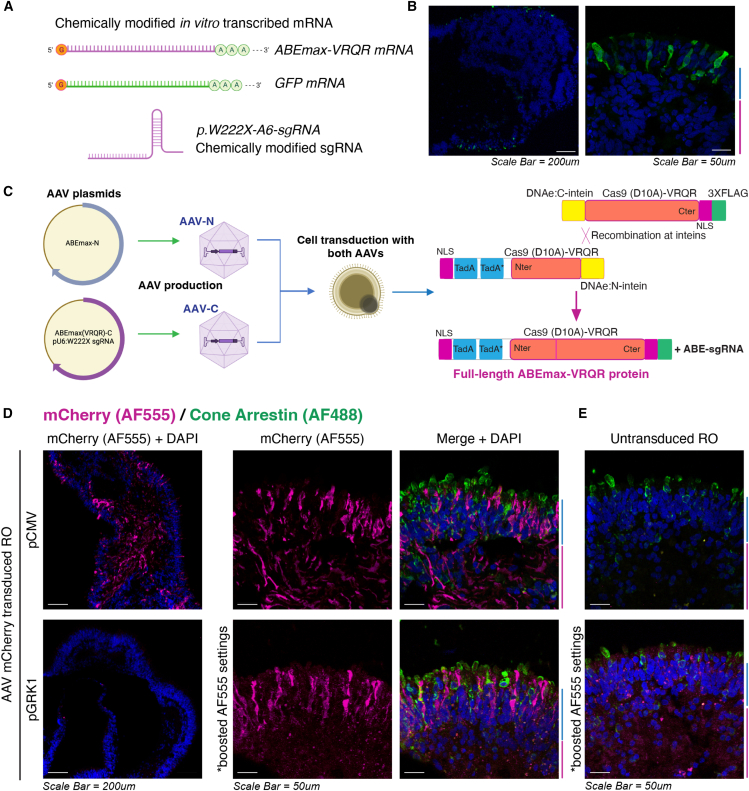
Characterization of mRNA/sgRNA lipofection and dual-AAV mediated delivery of ABE components to retinal organoids (A) Schematic diagram of ABE mRNA/sgRNA components for lipofection of ROs. Lipofection of ROs with chemically modified mRNA (encoding ABEmax-VRQR and GFP) and AIPL1 c.665G>A-A6-sgRNA was performed to achieve transient expression of base editor components in retinal organoid cells, with GFP mRNA used as a marker of lipofection efficiency. (B) GFP ICC of RO sections from W6.5 (D45) ROs collected 24 h after lipofection with 500 ng GFP mRNA. GFP-positive cells are largely limited to the photoreceptor precursors located within the laminated outer surface of the ROs. (C) Schematic diagram of the dual-AAV split intein system. The coding sequence for ABEmax-VRQR is split between two AAV plasmids, appended with N and C intein recombination peptide sequences. In RO cells successfully transduced with both AAVs, full-length ABEmax-VRQR is generated through intein *trans*-splicing and scarless protein recombination at the intein sites. (D) mCherry expression in ROs transduced with either pCMV-mCherry or pGRK-mCherry AAV (1 × 10^11^ vgc) (AAV2.7m8 serotype). ICC of RO sections for mCherry (red) and cone arrestin (green) reveals a widespread distribution of mCherry expression through the cell layers of AAV-pCMV-mCherry-transduced ROs, while AAV-pGRK1-mCherry drives expression only in the ONL/photoreceptor cell layer. Imaging settings were boosted to clearly show the AF555 signal in AAV-pGRK1-mCherry-transduced ROs, indicating lower levels of mCherry expression from the pGRK promoter compared to AAV-pCMV-mCherry-transduced cells. Images of untransduced RO sections imaged using the same settings are shown in (E). ONL and INL regions (blue and red bars, respectively) are demarcated. DAPI in blue. Scale bars, 50 μm.

Having established that ABE can correct c.665A>G, p.Trp222∗ in LCA4 patient iPSCs, two different delivery strategies were considered to test ABE in LCA4 RO models; lipofection of chemically modified ABE mRNA and sgRNA (Figures 3A and 3B), or RO transduction using the dual-AAV split-intein system (Figure 3C). Transient expression of ABE components is desirable due to the potential reduction in unwanted off-target editing; however, mRNA lipofection efficiencies and conditions are not well characterized for photoreceptor cells. Therefore, preliminary tests were conducted with GFP mRNA lipofection using commercially available reagents (STEMFect, Lipofectamine MessengerMax). Lipofection of mature ROs (after ∼ D160 of differentiation, when photoreceptor inner and outer segments have formed) showed no GFP positivity in photoreceptor cells on the RO surface (data not shown) under the conditions tested. In contrast, successful delivery of GFP mRNA was achieved with younger ROs, with GFP-positive photoreceptor precursors observed on the apical RO surface in lipofected ROs aged between D42 and D98 (Figure 3B). The ability to lipofect photoreceptor-lineage cells with these reagents therefore appears to decrease with increasing RO maturity.

For the dual-AAV split-intein system (Figure 3C), pCMV and pGRK1 (human rhodopsin kinase) promoter versions of the ABEmax-N and ABEmaxVRQR-C constructs were created to drive expression constitutively and specifically in photoreceptor-lineage cells, respectively. AAVs were assembled with the AAV7m8 capsid serotype (AAV2.7m8), which displays high tropism for retinal lineage cells, including photoreceptors.^36^ To better examine the activity of these promoters in RO cell populations, D210 ROs were transduced with AAV2.mCherry vectors (7m8 capsid) engineered with either pCMV or pGRK1 promoters (Figures 3D and 3E). Immunocytochemistry (ICC) for mCherry in RO sections revealed a widespread pattern of expression throughout RO cell populations for AAV-pCMV-mCherry, as expected for a constitutively active promoter. In contrast, AAV-pGRK1-mCherry transduced ROs displayed mCherry localization only in PR cells within the ONL, as expected from a PR-lineage promoter. The mCherry signal was notably weaker in AAV-pGRK1-mCherry-transduced RO cells (Figures 3D and 3E).

### Delivery of base editing components to LCA4 ROs can restore AIPL1 expression in photoreceptors

To assess the efficiency of corrective base editing following lipofection or transduction with the dual-AAV split-intein system, LCA4 model ROs (LCA4 patient and Trp222∗ KI) were treated at the appropriate time points and examined for AIPL1 immunoreactivity. For lipofections, developing ROs were treated with two rounds of lipofection a week apart, with treatment grouped into two conditions: early (first lipofection at ∼ D46) or late (first lipofection at ∼ D67) to assess whether the efficiency of lipofection and base editing differs during this RO developmental time window. For dual-AAV treatments, ∼D154 developmentally mature ROs with visible brush borders (consisting of apically protruding photoreceptor inner and outer segments) were treated and cultured for an additional 4 weeks to allow for AAV vector expression. Analysis of ROs for TUNEL positivity did not reveal altered levels of cell death or apoptosis in untreated versus treated ROs (Figure S5). Therefore, delivery of ABE components by either lipofection or viral transduction did not increase cell death or apoptosis.

**Figure 4:**
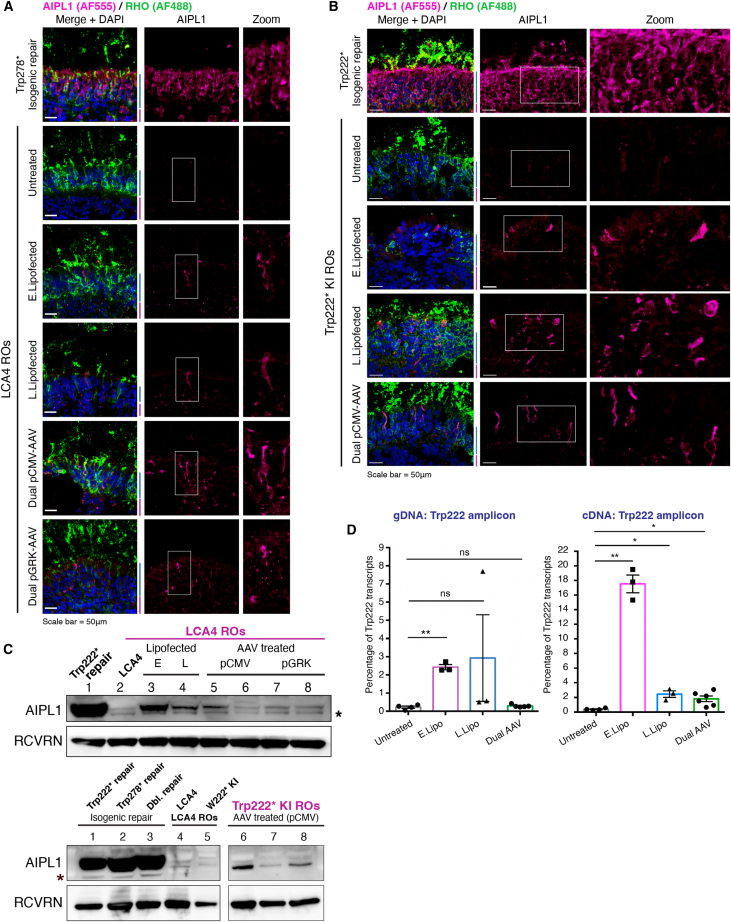
Both mRNA/sgRNA lipofection and dual-AAV-mediated delivery of base editing components rescue AIPL1 protein expression in LCA4 retinal organoids (A) ICC images of D255 sections from Trp278∗ isogenic repair and LCA4 patient ROs stained for AIPL1 (red) and rhodopsin (green). In isogenic Trp278∗ repair ROs, AIPL1 immunostaining is visible throughout the ONL, whereas it is absent in untreated LCA4 ROs. Rescue of AIPL1 expression can be seen in a subset of ONL cells in lipofected (early [E] and late [L]) and dual-AAV-treated (AAV2.7m8 serotype) LCA4 ROs. ABE was notably less efficient with pGRK1-AAV than with pCMV-AAV, with only a few cells showing AIPL1 positivity. DAPI in blue. Scale bars, 50 μm. (B) ICC images of D180 sections from Trp222∗ isogenic repair and Trp222∗ KI ROs treated with different ABE delivery methods and stained for AIPL1 (red) and rhodopsin (green). In ROs treated with either mRNA/sgRNA lipofection (early [E] and late [L]) or dual-AAV (pCMV) transduction (AAV2.7m8 serotype), AIPL1-positive cells can be seen in a subset of cells in the ONL of treated ROs. DAPI in blue. Scale bars, 50 μm. (C) Rescue of AIPL1 in treated LCA4 and Trp222∗ KI ROs can be detected by western blot analysis of whole RO protein extracts. Bands for AIPL1 are present in whole organoid protein extracts from LCA4 ROs and Trp222∗ KI ROs that were treated with mRNA/sgRNA lipofection and dual pCMV-AAV transduction (AAV2.7m8 serotype). AIPL1 protein levels appeared to be weak in dual pGRK1-AAV treated LCA4 ROs. A strong signal for AIPL1 was seen for all isogenic repair lines (Trp222∗ repair, Trp278∗ repair, and Trp222∗/Trp278∗ double repair). Recoverin (RCVRN) was used as a photoreceptor-specific loading control. ∗Nonspecific band. (D) HTS of the c. 665G>A, p.Trp222∗ region of Trp222∗ KI untreated and treated ROs (W26/D180). Genomic DNA and cDNA from ROs were analyzed for the occurrence of Trp222 compared to Trp222∗ to determine base editing frequency in samples. While nonsignificant changes were found between groups at the gDNA level (except for early lipofected ROs), there was a significant increase in Trp222 levels in all treated RO groups at the cDNA (transcript) level. Graphs show mean ± SD. Two-tailed parametric *t* test, Welch’s correction, *n* = 3 to 6 ROs per condition. ∗*p* < 0.05, ∗∗*p* < 0.01.

Age-matched control and treated ROs were analyzed for AIPL1 immunoreactivity. In LCA4 patient ROs (compound heterozygous for p.Trp222∗ and p.Trp278∗) treated at both lipofection time points, ICC for AIPL1 revealed AIPL1-positive cells (observed as single cells or clusters) in the ONL, indicating that *AIPL1* c.665G>A, p.Trp222∗ was successfully base edited in progenitor cells and that these cells differentiated to give rise to AIPL1-expressing photoreceptors (Figure 4A). For dual-AAV transduced LCA4 ROs, similar patterns of AIPL1-positive cells were present in the ONL of pCMV dual-AAV-treated ROs but were sparser in pGRK1 dual-AAV ROs treated with the same number of viral particles, indicating that pGRK1-driven expression of split-intein ABE components was less efficient (Figure 4A). For Trp222∗ KI ROs (homozygous for p.Trp222∗), the same lipofection conditions and pCMV dual-AAV treatment yielded similar patterns of AIPL1 rescue within the ONL PR cell population in treated ROs (Figure 4B). The restoration of AIPL1 protein (∼43 kDa) to varying levels in both LCA4 and Trp222∗ KI ROs treated by both lipofection and pCMV dual-AAV transduction was detected by western blot analysis (Figure 4C). Specific AIPL1 bands were barely detectable in untreated LCA4 and Trp222∗ KI RO samples, whereas a very strong signal for AIPL1 was present in all isogenic repair RO extracts. To quantify the degree of ABE in ROs, HTS was conducted on both gDNA and cDNA from whole Trp222∗ KI ROs to determine the percentage of corrected reads (Figure 4D). Interestingly, at the gDNA level, significant ABE was only detected in early lipofected ROs (2.42% editing), while analysis of cDNA transcripts showed a significant level of editing for all treated groups, with the highest editing observed in the early lipofected group (17.5% editing). The enhanced level of ABE seen at the cDNA level is expected, given the relatively higher expression of retinal cell type-specific transcripts, including the photoreceptor-specific expression of *AIPL1*. While there are indications of differing efficiencies, these results indicate that both mRNA/sgRNA lipofection and pCMV dual-AAV transduction can deliver the components necessary to drive corrective ABE in LCA4 model RO photoreceptors and restore AIPL1 protein expression.

### Rescue of rod phosphodiesterase subunits in treated ROs

**Figure 5:**
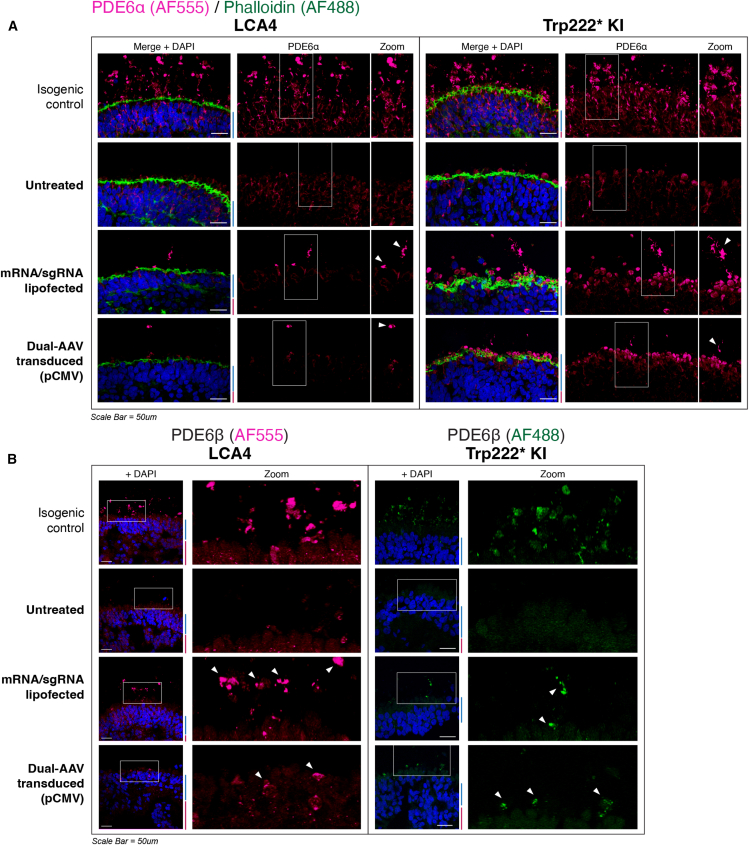
Treatment of LCA4 and Trp222∗ KI retinal organoids with adenine base editing components can restore rod PDE6 in some photoreceptors (A) ICC of RO sections for the PDE6 holoenzyme subunit PDE6α (red). In both LCA4 (D255) and Trp222∗ KI (D180) ROs, there is a loss of strong PDE6α signal in the photoreceptor outer segments (OSs), compared to staining observed in isogenic control ROs. Both mRNA/sgRNA lipofection and dual-AAV (AAV2.7m8 serotype) treatment resulted in rescue of this phenotype in a small subset of PR cells. RO sections were co-labelled with phalloidin-AF488 (green) to highlight the outer limiting membrane (OLM). DAPI in blue. Scale bars, 50 μm. (B) ICC of RO sections for the PDE6 subunit PDE6β. Abundant PDE6β is observed in the OS regions of isogenic heterozygous repair photoreceptor cells. LCA4 (D255) and Trp222∗ KI (D180) ROs display a similar pattern of PDE6β loss in the OS regions, with rescue of PDE6β in OSs observed in a subset of photoreceptor cells in treated ROs (positive OSs demarcated by white arrowheads). DAPI in blue. Scale bars, 50 μm. For (A) and (B), all isogenic control RO images shown are from Trp222∗ repair RO sections. mRNA/sgRNA lipofected RO images are of early lipofected ROs.

AIPL1 functions as a co-chaperone protein that, together with HSP90, is essential for the assembly of the cyclic GMP phosphodiesterase 6 (cGMP PDE6) holoenzyme complex, comprising PDE6α, PDE6, and the inhibitory PDE6γ subunits in rod photoreceptors.^37^^,^^38^^,^^39^ PDE6 is a critical component of the phototransduction cascade in rods, catalyzing the hydrolysis of cGMP in response to light stimulus. We have previously demonstrated that LCA4 RO rod photoreceptors have vastly reduced levels of PDE6α and PDE6β proteins and increased levels of cGMP.^27^^,^^35^^,^^36^ In isogenic repair organoids, high levels of PDE6α protein were predominantly localized to the outer segment (OS) regions of rods, while in LCA4 model organoids (LCA4 patient and Trp222∗ KI), PDE6α levels were greatly reduced but still detectable in the cell body of rod photoreceptors (Figure 5A). The expression and OS localization of PDE6α was restored in some photoreceptors in both lipofected and dual-AAV treated ROs (Figure 5A). PDE6β localization appeared exclusively in the rod OS region of isogenic repair ROs, and immunostaining for PDE6β was undetectable in LCA4 model photoreceptors. Similar to PDE6α, restoration of PDE6β immunostaining in the OS region was evident in some PR cells in treated LCA4 model ROs (Figure 5B). These results indicate that successful base editing of *AIPL1* c.665G>A, p.Trp222∗ is sufficient to restore downstream AIPL1 functionality in rod-lineage photoreceptors.

### ABE-treated LCA4 model ROs exhibit reduced levels of cGMP

**Figure 6:**
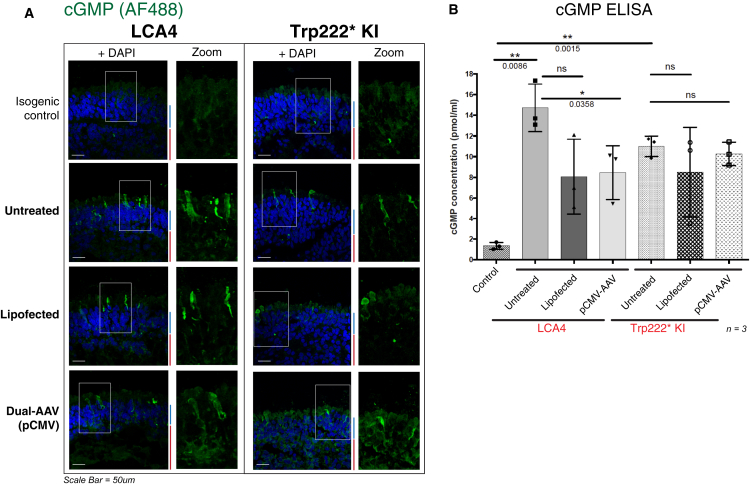
Measurement of cGMP levels in LCA4 model retinal organoids (A) ICC of cGMP (green) in untreated and treated (lipofected/dual-AAV transduced [AAV2.7m8 serotype]) LCA4 (D255) and Trp222∗ KI (D180) RO sections. cGMP levels are elevated in both LCA4 models, with subsets of photoreceptor cells in the ONL strongly positive for cGMP. cGMP-positive cells are also observed in treated RO sections. The isogenic control RO sections are from heterozygous repair lines Trp278∗ repair and Trp222∗ repair for the LCA4 and Trp222∗ KI panels, respectively. (B) Determination of cGMP concentrations in D160 whole LCA4 and Trp222∗ KI ROs (treated and untreated) by cGMP ELISA. cGMP levels were normalized to laminated RO perimeter. Both untreated LCA4 and Trp222∗ KI ROs display significantly elevated cGMP levels compared to control ROs. Analysis of cGMP levels in treated ROs showed significantly decreased cGMP levels only in dual-AAV transduced LCA4 ROs. Graph shows mean ± SD. Two-tailed parametric *t* test with Welch’s correction, *n* = 3 RO per condition. ∗*p* < 0.05, ∗∗*p* <0.01.

We have previously characterized the pattern of cGMP distribution in LCA4 loss-of-function RO models and showed that levels are strongly elevated compared to the very low baseline levels observed in wild-type ROs—an expected finding given the loss of PDE6 activity in the absence of functional AIPL1.^27^^,^^35^^,^^36^ Interestingly, this increase in cGMP levels is not evenly distributed throughout photoreceptor populations in ROs but is instead localized to isolated cGMP-positive photoreceptor cells in LCA4 ROs. To investigate whether ABE treatment of LCA4 model ROs affects cGMP levels, ICC was conducted to examine the distribution of cGMP-positive cells in ROs (Figure 6A). cGMP levels were undetectable in isogenic control ROs, while isolated populations of cGMP-positive cells were present in the ONL of LCA4 model ROs (LCA4 and Trp222∗ KI, untreated). cGMP-positive cells were similarly detected in both mRNA/sgRNA lipofected and dual-AAV treated ROs (Figure 6A). Quantitation of cGMP levels was conducted by cGMP ELISA analysis of whole RO extracts (Figure 6B). Untreated LCA4 patients and Trp222∗ KI ROs displayed significantly elevated cGMP levels compared to control ROs. While reduced cGMP levels were detected in lipofected and dual-AAV-transduced ROs in both models, this decrease was significant only in dual-AAV-transduced LCA4 patient ROs, which exhibited approximately a 50% reduction in cGMP levels (Figure 6B).

## Discussion

In this study, we investigated a panel of patient IRD variations for successful ABE in patient iPSCs and tested the most successful editor in ROs. To date, successful ABE has been reported for several IRD variations in *RPE65, KCNJ1, PDE6B, RHO, USH2A,* and *ABCA4*. Three independent studies reported successful ABE of *Rpe65* c.130C>T, p.Arg44∗ in the *rd12* mouse model.^12^^,^^13^^,^^14^ All three studies optimized ABE parameters against the mouse *Rpe65* target sequence, followed by *in vivo* investigations in the *rd12* model involving delivery of the base editors to the RPE via subretinal lentiviral (ABEmax, NG-ABE) or dual-AAV injections (split NG-ABE or NG-ABEmax). Similarly, two studies investigated ABE of *Pde6b* c.1678C>T, p.R560C in the *rd10* model.^15^^,^^16^ *In vitro* ABE optimization was conducted against the mouse *Pde6* target sequence, followed by dual-AAV-mediated (split NG-ABE8e or SpRY-ABE8e) subretinal delivery to postmitotic photoreceptors in *rd10* mice. A similar approach investigated ABE correction of mouse *Rho* p.Gln344∗, p.Thr17Met, and p.Glu150Lys *in vitro* and *in vivo* in mice via subretinal delivery of dual-AAV (split ABE8e and ABEmax).^17^^,^^18^ *In vivo* DNA editing efficiencies varied widely across these studies (∼10%–∼30%), but all showed positive outcomes for the rescue of retinal structure and function. Given the lack of conservation between mouse and human sequences, our study focused on patient-derived iPSCs and human-relevant ROs. A few other studies have investigated ABE efficiency in the human sequence context. Kabra et al. (2023) used RPE-targeting PEG-conjugated silica nanocapsules (SNC) to encapsulate and deliver ABE8e mRNA and sgRNA targeting *KCNJ13* c.158G>A, p.Trp53∗ to patient fibroblasts and iPSC-RPE.^19^ *In vivo* ABE was investigated in a monoallelic KI mouse model harboring the human variation, with delivery of ABE8e mRNA and sgRNA packaged into RPE-targeting SNC-PEG-all-trans retinoic acid (ATRA). For editing in postmitotic photoreceptor cells, Tachida and colleagues generated *AAVS1*-inserted HEK293T *USH2A* transgene stable cell lines to interrogate ABE of 35 *USH2A* variations and further investigated *USH2A* c.11864G>A, p.Trp3955∗ in a humanized KI mouse model.^20^ Dual-AAV-mediated delivery of split ABE8e targeting this variation showed precise editing efficiency of ∼53.5% and local restoration of USH2A localization in photoreceptors. Similarly, He et al. (2025) developed a humanized KI mouse model incorporating a DNA segment with the human *RHO* c.C50T, p.Thr17Met variation.^21^ *In vitro* optimization was first conducted using a *RHO* c.C50T, p.Thr17Met HEK293T cell line, and subretinal dual-AAV delivery of split ABE8e resulted in an editing efficiency of ∼27%, leading to rescue of retinal and visual function in mice. The use of patient iPSCs and human ROs circumvents the need to develop humanized mouse models. To date, the efficiency of ABE editing in ROs has been investigated in only one other study.^22^ ABE targeting *ABCA4* c.5882G>A, p.Gly1961Glu was conducted *in vitro* in a HEK293T cell line carrying the variation in a lentivirus insert, in human ROs, and *in vivo* in a humanized *Abca4* mouse model.^22^ Moreover, synonymous bystander editing immediately adjacent to the target adenine was used as a surrogate readout for editing of the locus in human iPSC-RPE, retinal and RPE/choroid explants, and in nonhuman primates (NHPs). An engineered split ABE (ABE8.5-m),^7^ optimized *in vitro* in the HEK293T model, resulted in a low percentage of A-to-G conversion (<5%) in ROs at both on-target and bystander adenines following dual-AAV-mediated delivery. Optimization of the AAV vector components significantly improved the editing efficiency in ROs by at least 3-fold. Notably, higher DNA editing efficiencies were achieved in the *Abca4* mouse carrying the humanized *Abca4* c.5882G>A allele (∼31% in the retina), and the highest DNA editing at the adjacent bystander site in NHPs (∼18% in the retina) was achieved with the highest dose (3 × 10^11^ v.g.) of virus (AAV5 serotype) tested.^22^

In this study, we identified an IRD-associated gene variant, *AIPL1* c.665G>A, p.Trp222∗, that is highly amenable to correction by ABE in patient iPSCs. We further demonstrate that base editing can occur in the target cell type through delivery of base editing components to cells in a patient-derived RO model, resulting in restoration of AIPL1 protein in subsets of photoreceptors. While we attribute the correction of AIPL1 c.665G>A, p.Trp222∗ and recovery of full-length AIPL directly to ABE, treatment with the editing system lacking sgRNA or with sgRNA alone could be useful to identify any incidental effects in treated ROs; however, we did not detect any other morphological changes or increased cell death in our treated ROs. Moreover, while both mRNA/sgRNA lipofection and dual-AAV delivery were able to achieve some degree of correction of *AIPL1* c.665G>A in the RO model under the conditions tested, the efficiency was relatively limited. Subsets of photoreceptors displayed restored AIPL1 and downstream PDE6α and PDE6β expression, but a consistent impact on whole-RO cGMP levels—one of the molecular hallmarks of LCA4 pathology—was not observed. To overcome this, the efficiency of base editing and ABE delivery would need to improve, or alternative delivery methods should be investigated. Of the two systems tested in this study, each delivery technique exhibited specific limitations.

Overall, the highest editing efficiencies were observed in immature lipofected ROs, highlighting the promise of mRNA/sgRNA lipofection as a delivery technique for achieving gene editing. However, late-stage, developmentally more mature ROs with established photoreceptor brush borders were more resistant to lipofection in the photoreceptor population under the same conditions. Tailored reagents for mRNA/sgRNA packaging to facilitate lipofection of mature PRs would improve the translatability of this work to an *in vivo* setting. Interestingly, recent studies describe lipid nanoparticles (LNPs) with specific modifications, such as PEG or peptide conjugation, to enhance the mRNA lipofection efficiency of retinal cells *in vivo* in mouse and NHP models,^40^^,^^41^ with robust lipofection of photoreceptors and other cell types observed after subretinal injection. It remains unknown whether RO models are similarly amenable to lipofection with these LNP reagents.

Even though ABEs have a favorable safety profile, transient expression of the editor—such as that achieved after nonviral mRNA delivery, compared to prolonged expression following AAV delivery—is considered ideal from a safety standpoint when evaluating approaches for the delivery of base editing components. For *AIPL1* c.665G>A, p.Trp222∗, a small amount of potentially deleterious bystander editing at A3 was observed at the target locus by HTS, and a level of editing was also detected at one predicted off-target site (predicted to have no effect on gene expression or function). Long-term expression of the base editor and sgRNA would likely result in cumulative increases in unintended off-target editing over time.

An alternative approach to achieving transient base editor expression is through delivery in the form of ribonucleoproteins (RNPs), which has yet to be demonstrated in photoreceptors but has been successful in RPE *in vivo*. Delivery of base editor-sgRNA RNPs within a retroviral protein scaffold has been achieved using engineered viral-like particles, resulting in correction of *RPE65* p.Arg44∗ within the RPE in the *rd12* LCA mouse model.^11^ Some degree of correction in the same mouse model has also been demonstrated using RNPs (for both base and prime editors) encapsulated in optimized LNPs.^42^ The use of RNPs may result in faster editing kinetics compared to mRNA delivery by bypassing the mRNA translation step.

In our *in vitro* RO model, further optimization of the dual-AAV system will be necessary to achieve higher levels of editing. Muller et al. highlight the critical importance of split-site placement within the Cas9 protein sequence, the number of NLS sequences, and the split-intein type, and polyA selection on editing efficiency.^22^ An ABE variant split at position 310 of SpCas9 showed the highest editing efficiency *in vitro* in a lenti-*ABCA4*^1961E^ HEK293T line targeting *ABCA4* c.5882G>A, p.Gly1961Glu. Use of the split-consensus fast DnaE (Cfa) intein, two bipartite NLSs per ABE half, and the bovine growth hormone (bGH) polyA, truncated simian virus 40 (trunc SV40) polyA, or truncated woodchuck hepatitis virus posttranscriptional regulatory element (WPRE) late SV40 (W3-late SV40) polyA all significantly increased editing efficiencies in ROs. In addition, improving the efficiency of AAV packaging to minimize empty and partial AAV genomes can have a major impact, influencing cell toxicity and immunogenicity in response to AAV dosage. Alterations to the AAV manufacturing process and improvements to the ABE split-intein system—which in our study utilized Npu intein sites and only two bpNLS motifs—may improve both transduction and ABE reconstitution efficiencies, thereby boosting overall editing levels in the RO system.

Finally, for base editing of IRD variations in ROs, optimal base editor selection for each genomic locus of interest will depend on criteria such as PAM site specificity, editing efficiency, and off-target or bystander editing. The development of compact base editors based on alternative or highly altered Cas proteins that can be encoded by a single AAV opens the possibility of more effective single-AAV-mediated base editing and potentially enables targeting of sites resistant to editing with SpCas9-family base editors.^43^^,^^44^^,^^45^ Additionally, engineering next-generation ABEs wih more active deaminase domains has significantly increased the potency of these base editors,^6^^,^^7^^,^^46^ potentially enhancing their ability to rescue IRD variants. Of the four IRD-associated genetic variants examined in this study as candidates for ABE, only *AIPL1* c.665G>A, p.Trp222∗ proved to be an ideal candidate for base editing when editing efficiency and off-target effects were assessed with various ABE-sgRNA and ABEmax-family base editor combinations. With the exception of *AIPL1* c.834G>A, p.Trp278∗—which displayed significant rates of deleterious bystander adenine editing—no or negligible levels of editing were observed at other sites. ABE8e is the most efficient ABE to date, has been shown to exhibit drastic improvements in editing efficiency and deamination rates (>500-fold) compared to its predecessor, ABE7.10.^46^ It is possible that the *RHO* and *RP2* variants tested in this study could be amenable to efficient editing by ABE8e. Moreover, resistance to editing and deleterious bystander effects could potentially be circumvented through testing of alternative base editors, some of which feature expanded or shifted editing windows and alternative PAM site specificities. Alternatively, prime editing can also elicit precise gene edits without creating DSBs.^2^ This approach may allow targeting of IRD-associated genetic variants that are resistant to base editing or where the target adenine is situated in an ABE editing window containing other adenines.

In conclusion, we show that ABE correction of the IRD-associated *AIPL1* variant c.665G>A, p.Trp222∗ can restore AIPL1 expression in a human RO model. However, while ROs modeling IRDs serve as powerful tools for the *in vitro* validation of corrective base editing, achieving specific and efficient editing of G-to-A or C-to-T transitions associated with IRDs may require significant optimization of ABE parameters.

## Materials and methods

### Base editing plasmids

Details of the plasmids used for base editing experiments are provided in Table S5. For cloning ABE-sgRNAs into the sgRNA expression plasmid pSPgRNA, oligonucleotides corresponding to the 20-nt sgRNA protospacer sequences were flanked by overhangs (top strand: 5′-CACCG-[*20-nt protospacer*]-3′; bottom strand: 5′-AAAC-[*20-nt reverse complement sequence*]-C-3′). Protospacer sequences are listed in Table S2. Oligonucleotides were phosphorylated with T4 polynucleotide kinase (New England Biolabs, NEB, Uxbridge, UK), heated to 96°C, and slowly annealed to form double-stranded oligonucleotide fragments with the requisite sticky ends for ligation into the *BbsI*-linearized plasmid backbone.

Expression plasmids for ABEmax and its variants (VRQR, NG, and SpRY) were tested. The split-intein plasmids were constructed through multiple steps. AAV2-pCMV-ABEmax-N was generated by PCR amplification of the ABEmax N terminus (AA 1–987) from pCMV-ABEmax-P2A-GFP, and the N-intein fragment from pLV302. The 5′ end of the N-intein forward primer included 30 nt of sequence complementary to the 3′ end of ABEmax-N. Both fragments were fused by overlap extension PCR, digested with *AgeI* and *SphI* restriction enzymes (restriction sites introduced via the primers), and cloned into the *AgeI*- and *SphI*-linearized AAV2-ITR plasmid pLV302, which carries the CMV promoter. For the construction of AAV2-pCMV-ABEmaxVRQR-C:U6-sgRNA, primers were designed to amplify ABEmax-C (AA 988–1806) from pCMV-ABEmax-P2A-GFP and the C-intein sequence from pLV312.3. The primer design incorporated a 3×FLAG tag at the 3′ end of ABEmax-C and eb=nabled overlap extension PCR to generate a fusion product flanked by *NcoI* and *PacI* restriction sites. This digested product was cloned into *NcoI*- and *PacI*-linearized pLV312.3. The pU6-*S. pyogenes* sgRNA expression cassette was then engineered into this intermediate plasmid by amplifying the region from pSPgRNA, and ligating it into the *KpnI*- and *NotI*-linearized plasmid backbone to form AAV2-pCMV-ABEmax-C:U6-sgRNA. The ABEmax-VRQR version was subsequently generated by cloning the *EcoRV*+*EcoRI* VRQR-specific region from pCMV-ABEmax-VRQR into the plasmid backbone linearized with the same enzymes. Finally, p.Trp222∗-A6-ABE-sgRNA was cloned into AAV2-pCMV-ABEmax-C:U6-sgRNA following the same procedure as for pSPgRNA, using *BbsI*-linearization of the construct to allow ligation of the annealed spacer oligos.

For the pGRK1 variants of the split intein plasmids, primers were designed to amplify 326bp of the human GRK1 promoter region (encompassing the previously characterized photoreceptor-specific −112 to +183 region)^47^ and to incorporate flanking *XhoI* and *AgeI* restriction sites. The digested product was ligated into the N- and C-terminal split-intein construct backbones, linearized with *XhoI*/*AgeI* (pCMV excised).

pCMV and pGRK1 AAV2-mCherry plasmids were generated using an NEBuilder (NEB) cloning strategy. Primers were designed to amplify the mCherry-SV40 polyA region from pmCherry (Takara Bio Europe Ltd., Uxbridge, UK), with 20 nt homology arms incorporated at the 5′ and 3′ ends for scarless integration into *AgeI*/*NotI*-linearized AAV2 plasmids (both pCMV and pGRK1 versions). AAV plasmids were propagated in NEB Stable Competent *E. coli* (High Efficiency) DH5 alpha cells according to the manufacturer’s guidelines. Primer sequences for the cloning of all constructs are provided in Table S6.

### Human iPSC and RO culture

Patient iPSC lines used in this include LCA4-1, LCA4-4, RHO-p.Pro347Leu, and RP2-p.Arg120∗.^26^^,^^27^ Primers for screening genomic DNA at all these loci are listed in Table S7 iPSCs were maintained in mTESR Plus (STEMCELL Technologies, Oxford, UK) on Geltrex (Gibco, Romsey, UK)-coated plates, with passaging every 5–7 days. ROs were established as previously described,^27^ with modification to some sets of RO differentiations from D100 onwards to enhance outer segment formation. Instead of the standard medium, ROs were cultured in Advanced DMEM/F12 (Gibco) supplemented with 10% fetal bovine serum (FBS, Gibco), 1% N2 Supplement (Gibco), 2% B27 supplement (without vitamin A), 100 uM taurine, 4 mM GlutaMAX (Gibco), 1.4 g/L glucose (Gibco), 1× Anti-Anti (Gibco), and 50 uM docosahexaenoic acid (DHA).

### Generation of isogenic iPSC lines

The P3 Primary Cell 4D-Nucleofector X Kit S (Lonza, Cambridge, UK) was used for all iPSC nucleofection experiments. iPSCs were cultured in Stemflex (Gibco) with 10 uM ROCK inhibitor Y-27632 (STEMCell Technologies) for 2 h prior to single-cell dissociation with TrypLE (Gibco). For each nucleofection sample, 2 × 10^5^ cells were used. For correction of *AIPL1* c.665G>A, p.Trp222∗, iPSCs were nucleofected with a mix of 1μg pCMV-ABEmax-VRQR and 0.5μg p.Trp222∗-sgRNA expression plasmids. To generate *AIPL1* c.665G>A, p. Trp222∗ homozygous cells, a 20 nt gRNA (NGG PAM) and 127 nt single-stranded oligo deoxynucleotide (ssODN) template were designed to incorporate c.665G>A, p.Trp222∗ into the heterozygous wild-type p.Trp222 allele via CRISPR-mediated HDR. The CRISPR RNA (crRNA) and ssODN, with phosphorothioate (PS) modifications at the ends, were obtained from Integrated DNA Technologies (IDT, Bristol, UK). Repair of AIPL1 c.834G>A, p.Trp278∗ was achieved using a previously characterized combination of ssODN and crRNA for CRISPR-mediated HDR of the locus.^27^ iPSC clones were derived from mixed nucleofected populations and screened to establish stable iPSC lines. Sequences for crRNAs and ssODNs are provided in Table S4.

### Chemically modified mRNA and sgRNA lipofection

3 nmol of chemically modified AIPL1-p.Trp222∗-A6-sgRNA (TrueGuide Synthetic gRNA) was obtained from Thermo Fisher Scientific (Romsey, UK) and resuspended to a concentration of 100 nmol/μl. For the synthesis of ABEmax-VRQR and GFP mRNA, the HiScribe T7 ARCA mRNA Kit (with tailing) (NEB) was used. 1 μg of *SapI*-linearized pCMV-ABEmax-VRQR plasmid/1μg of *SapI*-linearized pCMV-T7-EGFP was used as the templates, and the resulting mRNAs were polyA-tailed and purified via LiCl precipitation. Each lipofection (5–10 ROs) comprised 500-750 ng of ABEmax-VRQR mRNA, 250-300 ng of GFP mRNA, and 100 ng of A6-sgRNA. Commercially available lipofection reagents—Stemfect RNA Transfection Kit (ReproCELL, Wakefield, UK) or Lipofectamine MessengerMAX (Invitrogen, Paisley, UK)—were used according to the manufacturer’s instructions. ROs were incubated in a volume of 200–300 μL media with the lipofection mix added for a period of 3–5 h, with 1–2 mL of media added after. ROs were assessed for GFP positivity 24–48 h later. ROs were treated with a second round of lipofection one week later, following the same conditions.

### Generation of AAV and AAV transduction

AAV was produced from low-passage HEK293T cells through equimolar tri-plasmid transfection. The pAdDeltaF6 helper plasmid, 7m8 serotype plasmid, and AAV2-ITR plasmid contained the components for AAV production in cells (Table S5). For AAV production, 400–1000 ng of total plasmid DNA was used to transfect 8 × 15 cm^2^ plates of HEK cells at 70% confluency. Each plate was seeded with 8.9 × 10^6^ HEK cells 24 h prior. The calcium phosphate transfection method was used; a mix of 25 mL of water, freshly dissolved 0.25 M CaCl_2_, and plasmid DNA was added dropwise to a continually bubbling 25 mL solution of 2× HEPES buffered saline solution (HBSS). This mix was evenly distributed (6.25 mL/plate) among HEK cultures. Cultures were harvested after 65 h. AAV was purified either using the AAVpro (all serotypes) Maxi Purification Kit (Takara) for extraction from pelleted cells, or through chloroform purification (which allows for extraction of AAV from both cells and media), as detailed in Negrini et al.^48^ To determine the concentration of AAV preparations, 2 μL was taken for DNAseI treatment to remove contaminating DNA before qPCR analysis of serial dilutions of the AAV samples using primers specific for the AAV2-ITR sequences (Table S8).^49^ qPCRs were assembled with 2× LabTaq Green Hi Rox Master Mix (Labtech, Heathfield, UK) and conducted using an Applied Biosystems QuantStudio 6 Flex real-time PCR system.

For AAV transduction, 5–10 ROs (∼D154+, with developed photoreceptor brush borders) were incubated with 1 × 10^11^ vgc of each AAV in a volume of 150–300 μL of RO growth medium in low-cell-adherence-treated 96-well plate wells or tilted 25-well plate wells. 1.5–2 mL of media was added to each batch of ROs after 5 h. Half-media changes were conducted every 2 days, and ROs were cultured for at least another 4 weeks before analysis.

### Processing of DNA and RNA samples

The Wizard Plus SV Miniprep Kit (Promega, Cambridge, UK) was used to isolate DNA from iPSC samples. For RO samples, DNA was extracted using the same kit or via TRIzol (Thermo Fisher Scientific). Purification of RNA from ROs was achieved using the ARCTURUS PicoPure RNA Isolation Kit (Applied Biosystems) or via TRIzol. For TRIzol extractions, ROs were processed in 0.3 mL TRIzol (with subsequent reagents scaled down accordingly), following the manufacturer’s guidelines. cDNA synthesis from RNA samples was conducted using the High-Capacity cDNA Reverse Transcription Kit (Applied Biosystems, Reading, UK) with random hexamers.

### Sanger sequencing and EditR analysis

Primers for PCR amplification are listed in Table S7. PCR amplification was carried out using either GoTaq Green master mix (Promega) or Q5 High-Fidelity 2× Master Mix (NEB), with primers at a concentration of 0.25 pmol/μl. PCR samples were cleaned by gel extraction (Monarch DNA Gel Extraction Kit, NEB) or plate purification (MultiScreen 96-well PCR Filter Plates, Millipore) before submission for Sanger sequencing analysis conducted by Source Bioscience. EditR (EditR: https://moriaritylab.shinyapps.io/editr_v10/) was used to calculate base editing rates from Sanger sequencing results.^31^

### Next-generation sequencing

Universal tagged primers for MiSeq (Illumina) high-throughput sequencing (HTS) are listed in Table S9. PCR amplification was carried out using High-Fidelity 2× Master Mix (NEB). Products were gel-extracted (Monarch DNA Gel Extraction Kit, NEB), and 300–500 ng of product was used for subsequent steps. Processing of the samples using the MiSeq Reagent Nano Kit v2 was carried out at the UCL Cancer Institute CAGE Facility. Analysis of HTS data was conducted with CRISPResso2 (CRISPResso2: http://crispresso2.pinellolab.org/submission) with stringent quality filtering and trimming specifications (minimum average read quality [phred33 scale]: >30; minimum single bp quality [phred33 scale]: >20; replace bases with “N” that have a quality lower than [phred33 scale]: <20; exclude bp from the left side of the amplicon sequence for the quantification of mutations: 15; exclude bp from the right side of the amplicon sequence for the quantification of mutations: 15; trimming adapter: no trimming).

### ICC and imaging

ROs were processed for ICC and imaging as previously described.^27^ Lists of primary and secondary antibodies and stains used are provided in Tables S10–S12. The In Situ Cell Death Detection Kit, Fluorescein (Roche, Mannheim, Germany), was used to stain for dead cells.

### Protein extraction

For HEK cell samples and some RO samples, cells were washed with DPBS and lysed on ice for 10–15 min in 40–100 μL cold RIPA buffer (50 mM Tris-HCL, pH 7.5; 150 mM NaCl; 1 mM EDTA; 1% NP-40; 0.5% sodium deoxycholate; 0.1% SDS) supplemented with 2% protease inhibitor cocktail (Sigma-Aldrich, Glemsford, UK). The samples were briefly sonicated and spun at 12K RPM for 10 min at 4°C to pellet insoluble particles in the lysate. Protein quantitation of cell lysates was performed using the Pierce BCA Protein Assay kit (Thermo Fisher Scientific) according to the manufacturer’s guidelines. For protein extracts from TRIzol-processed samples, the protein fraction was isolated and washed according to the manufacturer’s guidelines. The protein pellet was resuspended in 40 μL 1% SDS and quantitated by BCA assay.

### Western blot analysis

Protein samples (10–25 μg) were prepared for loading using NuPAGE MOPS SDS Buffer Kit components (Thermo Fisher Scientific). Samples were heated at 70°C for 10 min and then loaded onto mPAGE# 4%–20% Bis-Tris pre-cast gels (Millipore). Pageruler Plus protein ladder (Thermo Fisher Scientific) was used as a reference for protein size. Gels were run at 100–150 V for approximately 1 h. Protein transfer was carried out in cold transfer buffer containing 20% methanol, at 35 V for 1 h, onto nitrocellulose membranes. Membranes were incubated in blocking buffer (PBS +0.1% Tween 20 + 5% skimmed milk powder [Sigma-Aldrich]) overnight at 4°C. Primary and secondary antibodies (Tables S10 and S11) were diluted in blocking buffer before being added to the membranes. Incubations with fluorescence-conjugated antibodies were conducted under dark conditions. Membranes were washed 4 × 5 min (PBS +0.1% Tween 20) after each antibody incubation step. Clarity Max ECL substrate (Bio-Rad, East Molesey, UK) was used to visualize membranes stained with HRP-conjugated secondary antibodies. A ChemiDoc imaging system (Bio-Rad) was used to image the membranes.

### HSP90 ELISA assay

The HSP90 ELISA assay using recombinant HSP90 was conducted as described in Sacristan-Reviriego et al.^38^

### cGMP ELISA assay

ROs were processed as previously described using 96-well Cyclic GMP ELISA kits (Cayman Chemicals, Ann Arbor, MI, USA).^27^ Similarly sized ROs were chosen for analysis.

### Statistical analysis

For HTS and cGMP ELISA analyses, values from at least three biological replicates per sample/RO type were used for the calculations of group averages and standard deviations (SDs). Pairwise comparisons were carried out using two-tailed *t* tests (∗ ≤0.05 significance, ∗∗ ≤0.01 significance, ∗∗∗ ≤0.001 significance, annotated on the relevant graphs), with Welch’s correction where appropriate.

## Data and code availability

The data related to this study are fully documented in the paper or in the supplemental information.

## Acknowledgments

The authors gratefully acknowledge funding from the 10.13039/501100000265Medical Research Council (MR/P02582X/1), 10.13039/100002089Fight for Sight (5129/5130), 10.13039/501100017645Moorfields Eye Charity (R170015A), and the 10.13039/100010269Wellcome Trust (205041/Z/16/Z). This research was supported by the 10.13039/501100000272National Institute for Health and Care Research Biomedical Research Centre at 10.13039/501100012617Moorfields Eye Hospital and the UCL Institute of Ophthalmology. The authors thank the Cancer Genomics Engineering (CAGE) Facility, UCL Cancer Institute, for the MiSeq preparation and sequencing. The CAGE Facility is supported by the BRC, the Welton Foundation, and in part by the 10.13039/501100000289Cancer Research UK – UCL Center. The authors thank Rafael Baganha and Rui J. Nobre (Center for Neuroscience and Cell Biology, and ViraVector for Gene Transfer Core Facility, University of Coimbra, Coimbra, Portugal) and Luís Pereira de Almeida (Center for Neuroscience and Cell Biology, Faculty of Pharmacy, University of Coimbra, Coimbra, Portugal) for assistance with AAV manufacture. The authors thank Viswanathan Ramamurthy (Departments of Ophthalmology, Biochemistry, and Pharmaceutical and Pharmacological Sciences, West Virginia University, USA) for the AIPL1 antibody. The authors thank Addgene for plasmids listed in Table S5: pSPgRNA (Addgene plasmid # 47108) was a gift from Charles Gersbach; pCMV-ABEmax (Addgene plasmid # 112095); pCMV-VRQR-ABEmax (Addgene plasmid # 119811), pCMV-NG-ABEmax (Addgene plasmid # 124163), and pCMV-ABEmax-P2A-GFP (Addgene plasmid # 112101) were a gift from David Liu; pLV302 (Addgene plasmid # 119943) and pLV312.2 (Addgene plasmid # 119944) were a gift from Gerald Schwank; pCMV-T7-EGFP (BPK1098) (Addgene plasmid # 133962) and pCMV-T7-ABEmax(7.10)-SpRY-P2A-EGFP (Addgene plasmid # 140003) were a gift from Benjamin Kleinstiver; pAdDeltaF6 was a gift from James M. Wilson (Addgene plasmid # 112867); and 7m8 was a gift from John Flannery and David Schaffer (Addgene plasmid # 64839).

## Author contributions

Conceptualization, P.R.L.P., M.E.C., J.v.d.S.; formal analysis, A.L., P.R.L.P., A.S.-R., P.E.S., E.A.A., F.O.R., K.Z., R.G., K-L.H., and J.v.d.S.; funding acquisition, P.R.L.P., M.E.C., and J.v.d.S.; investigation, A.L., P.R.L.P., and A.S.-R.; methodology, A.L., P.R.L.P., A.S.-R., P.E.S., E.A.A., F.O.R., K.Z., R.G., K-L.H., and J.v.d.S.; project administration, J.v.d.S.; resources, M.E.C. and J.v.d.S.; supervision, J.v.d.S.; validation, A.L. and P.R.L.P.; visualization; A.L., J.v.d.S.; writing – original draft; A.L. and J.v.d.S.; writing – review and editing, A.L., P.R.L.P., A.S.-R., P.E.S., K.Z., R.G., K.-L.H., M.E.C., and J.v.d.S.

## Declaration of interests

M.E.C. has been invited to serve as an Editorial Board Member for *Molecular Therapy Nucleic Acids* (*MTNA*).

## Supplemental information

Document S1. Figures S1–S5 and Tables S1–S12

Document S2. Article plus supplemental information
