# Supplementary material for: Adenine base editor correction of pathogenic variations associated with inherited retinal dystrophy in patient iPSC and retinal organoids
Source: Mol Ther Nucleic Acids. 2025 Nov 17;36(4):102777. doi: 10.1016/j.omtn.2025.102777 (PMC12704276; doi:10.1016/j.omtn.2025.102777)
Supplement: Document S1. Figures S1–S5 and Tables S1–S12 [file mmc1.pdf]

OMTN, Volume *36*

Supplemental information

Adenine base editor correction of pathogenic variations associated with inherited retinal dystrophy in patient iPSC and retinal organoids

Amy Leung, Pedro R.L. Perdigão, Almudena Sacristan-Reviriego, Paul E. Sladen, Erika A. Aguzzi, Farah.O. Rezek, Kalliopi Ziaka, Rosellina Guarascio, Kwan-Leong Hau, Michael E. Cheetham, and Jacqueline van der Spuy

SUPPLEMENTAL INFORMATION

Table S1: IRD Genetic Variants Targeted for ABE

Gene	Coding sequence position and change	Genomic position (GRCh38)	rsID	Protein change
AIPL1	665G>A	chr17: 6,426,734	rs1912011058	p.Trp222*
AIPL1	834G>A	chr17: 6,425,781	rs62637014	p.Trp278*
RP2	358C>T	chrX: 46,853,731	rs104894927	p.Arg120*
RHO	1040C>T	chr3: 129,533,710	rs29001637	p.Pro347Leu

Table S2: ABE-sgRNA Information

Gene	Gene variant (cDNA)	Protein change	sgRNA spacer sequence (20nt)	Position of target adenine in relation to 5'-3' spacer	Orientation	GC %	PAM sequence	Potential bystander edit?	Base editor	Editing observed?
AIPL1	665G>A	Trp222*	GCAGT**a**GCTGAAGCTGGAGA	A6	Sense	55	AGA	No	ABEmax-VRQR	Yes
AIPL1	834G>A	Trp278*	GTG**a**AATGAGGCCGAGGCCA	A4	Sense	60	AGG	Yes	ABEmax	Yes, +bystander
AIPL1	834G>A	Trp278*	GTGTG**a**AATGAGGCCGAGGC	A6	Sense	60	CAA	Yes (A7)	ABEmax-SPRY	No
AIPL1	834G>A	Trp278*	GGTGTG**a**AATGAGGCCGAGG	A7	Sense	60	CCA	No	ABEmax-SPRY	No
RP2	358C>T	Arg120*	CTC**a**CACACGAAATTGTTGG	A4	Antisense	45	CAG	Yes (A6)	ABEmax-SPRY	No
RP2	358C>T	Arg120*	TCTC**a**CACACGAAATTGTTG	A5	Antisense	40	GCA	Yes (A7)	ABEmax-SPRY	No
RP2	358C>T	Arg120*	ATCTC**a**CACACGAAATTGTT	A6	Antisense	35	GGC	No	ABEmax-NG	No
RP2	358C>T	Arg120*	AATCTC**a**CACACGAAATTGT	A7	Antisense	35	TGG	No	ABEmax/ ABEmax-NG	No
RHO	1040C>T	Pro347Leu	TAGGCC**a**GGGCCACCTGGCT	A7	Antisense	70	CGT	No	ABEmax-NG	No

Table S3: Cas-OFFinder Analysis of *In Silico* Predicted Off Target *AIPL1* c.665G>A-A6-sgRNA Editing

Potential OT site:	Genomic location (GRCh38)	Target sequence	Subs	Bulge	Position of potential off-target site
Off target site 1	Chr1: 20710950	GCAGgAGCTGAAGCTtGAGATGA	2	0	Intron 3 of kinesin family member 17 (*KIF17*), transcript variant 2, mRNA. (from RefSeq NM_001122819)
Off target site 2	Chr2: 140826079	GCAGgAGCTGAAGCTGaAGACGA	2	0	Intron 31 of LDL receptor related protein 1B (*LRP1B*), mRNA. (from RefSeq NM_018557)
Off target site 3	Chr12: 131166053	GCAGcAGgTGAAGCTGGAGAGGA	2	0	Intron 1 of long intergenic non-protein coding RNA 1257 (*LINC01257*), long non-coding RNA. (from RefSeq NR_026670)
Off target site 4	Chr9: 97325128	GCAGgAGCTGgAGCTGGAGAAGA	2	0	Exon 14 of coiled-coil domain containing 180 (*CCDC180*), transcript variant 1, mRNA. (from RefSeq NM_020893)
Off target site 5	Chr18: 55161544	GaAGTAGCTGAgGCTGGAGAAGA	2	0	Not in a gene
Off target site 6	Chr1: 5017839	GCAGaAGCTGAAG**T**CTGGAGATGA	1	1	Not in a gene
Off target site 7	Chr17: 68468336	GtAGTAGCTGAAGCTG**T**GAGAGGA	1	1	Intron 1 of *ENSG00000267009*
Off target site 8	Chr8: 141016235	GCAaTAGC**A**TaAAGCTGGAGAAGA	2	1	Not in a gene
Off target site 9	Chr5: 180845625	GC**C**ttTAGCTGAAGCTGGAGAAGA	2	1	Not in a gene
Off target site 10	Chr1: 158186690	GCAGTAGCTGGAAGCTGcAGgAGA	2	1	Not in a gene

Table S4: crRNA and ssODN Sequences:

Desired change	crRNA (20nt)	Orientation	GC%	ssODN (complementary to crRNA)
p.Trp222> p.Trp222*	**AGAAGCCGTGGGAGGTGCAG**	Sense	65%	C*C*AGCACCTCATAGTACTCCTCCTTCTTCAGCAGGCACTGGCAGTAGTT GAGGATCAGAGTATTGATCATCTTCTCCAGCTTCAGCTACTGCACCTCCCA CGGCTTCTCCTGCCCAGGGAGAAGGT*C*
p.Trp278*> p.Trp278	**TCACGCAGAGGTGTGAAATG**	Sense	50%	T*C*CAGCAGCCTCAGCTCCCTGCGCACCGCCTTCTGCATGGACGGCTCC AGCTCCAGCACTTTCTGGAGGTCCGCCTTGGCCTCGGCTTCGTTCCACAC CTCTGCGTGAGCCCGGGCACGCACGTA*G*T

Table S5: Plasmid Information

sgRNA / ABE expression plasmids	Addgene ref.	Notes
pSPgRNA	47108	ABE-sgRNA protospacer oligos cloned into BbsI-linearised plasmid backbone
pCMV-ABEmax	112095	-
pCMV-ABEmax-VRQR	119811	SapI-linearisation to prepare template for mRNA synthesis
pCMV-ABEmax-NG	124163	-
pCMV-ABEmax-P2A-GFP	112101	-
pCMV-T7-ABEmax-SpRY-P2A-EGFP	140003	-
pCMV-ABEmax-VRQR-P2A-GFP	-	EcoRV+EcoRI digestion to excise VRQR region from pCMV-ABEmax-VRQR, cloned into EcoRV+EcoRI-linearised pCMV-ABEmax-P2A-GFP
pCMV-ABEmax-NG-P2A-GFP	-	EcoRV+EcoRI digestion to excise NG region from pCMV-ABEmax-NG, cloned into EcoRV+EcoRI-linearised pCMV-ABEmax-P2A-GFP

AAV2 split intein plasmids		
pLV302	119943	-
pLV312.3	119944	-
pCMV-ABEmax-N	-	See materials and methods for details of construction
pGRK-ABEmax-N	-	See materials and methods for details of construction
pCMV-ABEmax-C(VRQR)-p.W222X-ABE-sgRNA	-	See materials and methods for details of construction
pGRK-ABEmax-C(VRQR)-p.W222X-ABE-sgRNA	-	See materials and methods for details of construction

Plasmid for GFP mRNA synthesis		
pCMV-T7-EGFP (BPK1098)	133962	SapI-linearisation to prepare template for mRNA synthesis

AAV2 mCherry plasmids		
AAV2-pCMV-mCherry	-	Generated through NEBuilder cloning
AAV2-pGRK-mCherry	-	Generated through NEBuilder cloning

Plasmids for AAV assembly		
pAdDeltaF6	112867	-
7m8	64839	-

Table S6: Primers for AAV2 Cloning

AAV2-pCMV-ABEmax-N primers	
**ABEmaxN (~3100 bp)**	**Amplification from pCMV-ABEmax-P2A-GFP (Addgene #112101)**
ABEmaxN-AgeI-Fwd	taactaccggtgccaccatgaaacggacagcc
ABEmaxN-Rev	Ctcgattttcttgaagtagtcctctttcagc
**N-intein (~600 bp)**	**Amplification from pLV302 (Addgene #119943)**
N intein(ABEmaxN)-Fwd	gctgaaagaggactacttcaagaaaatcgagtgtcttagctacgagacagagattcttacag
BGH-SphII-Rev	ACATgcatgcctgctattctcttcccaatc
AAV2-pCMV-ABEmax-C:U6-sgRNA primers	
**ABEmaxC (~2500 bp)**	**Amplification from pCMV-ABEmax-P2A-GFP (Addgene #112101)**
pCMV-NcoI-Fwd	CATGccatggtgatgcggttttggca
C-intein(ABEmaxC)-Rev	ggagatttccacggagtcgaagcagcttgcgataaaaccgttcttcaagg
**C-intein (~600 bp)**	**Amplification from pLV312.3 (Addgene #119944)**
ABEmaxC-Fwd	tgcttcgactccgtggaaatctcc
ABEmaxC-3Flag-PacI-Rev	CCttaattaattaCTTATCGTCATCGTCTTTGTAATCAATATCATGATCCTTGTAGTCTCCGTCGTGGTCCTTATAGTCtccgcttccgactttcctcttcttc
**U6-gRNA (~400 bp)**	**Amplified from pSPgRNA (Addgene #47108)**
U6-KpnI-Fwd	CGGggtaccgagggcctatttcccatg
gRNA-NotI-Rev	ATAAGAATgcggccgcaaaaaaagcac
pGRK primers (345bp): Amplified from human gDNA	
XhoI_hGRK1_Fwd	TTTTCTCGAG-GGGCCCCAGAAGCCTGGTGGTTGTTTGTC
AgeI_hGRK1_Rev	TTTACCGGT-AGCGTTCACAGACAGAGCAGGCCTGACTG
AAV2 mCherry construct primers: Primers for amplification of mCherry-SV40-polyA from pmCherry	
pCMV_NEBmChF	agagctctctggctaactaccgg**CTAGCCACCATGGTGAGCAA**
pGRK_NEB_mChF	tgctctgtctgtgaacgcta**CTAGCCACCATGGTGAGCAA**
NEBuilder_mChR	ccatcactaggggttcctgc**CGCTTACAATTTACGCCTTAAGATAC**

Table S7: Primers for PCR Amplification and Sequencing of Genomic Loci

Gene	Gene variant (cDNA)	Protein change	Forward	Reverse	Size
AIPL1	c.665G>A	p.Trp222*	CGGCTGGGTGGAGACAAG	GAAGTGGCGCTGACTCTGG	369bp
AIPL1	c.834G>A	p.Trp278*	TTGAGGAAACCGAGGGATGG	CAATCGAACCAGAAGTGACCAGG	582bp
RP2	c.358C>T	p.Arg120*	CACTCTGCTACAGTTACCATTG	CCTGCATCTTTGAACTGGAAAG	260bp
RHO	c.1040C>T	p.Pro347Leu	AAGCCTCTTGCCTTCCAGTT	GTGACTTCGTTCATTCTGCAC	240bp

Table S8: Primers for AAV2 Real Time PCR Quantitation (Aurnhammer et al. 2012)

	Forward	Reverse
AAV2 ITR	GGAACCCCTAGTGATGGAGTT	CGGCCTCAGTGAGCGA

Table S9: Primers for High Throughput Sequencing (HTS)

HTS region:	Forward Miseq primer	Reverse Miseq primer	Amplicon size bp w/o tags	Amplicon size bp w/ tags
gDNA *AIPL1*-p.W222X	TCGTCGGCAGCGTCAGATGTGTATAAGAGACAG-**GAAGTGGCGCTGACTCTGG**	GTCTCGTGGGCTCGGAGATGTGTATAAGAGACAG-**GAGAATATCACTGGTGTGCTCC**	228	295
cDNA *AIPL1*-p.W222X	TCGTCGGCAGCGTCAGATGTGTATAAGAGACAG-**AGAGGGAAATCGGCTCTTCAAG**	GTCTCGTGGGCTCGGAGATGTGTATAAGAGACAG-**GGAGAATATCACTGGTGTGCTC**	224	291
Off target site 1	TCGTCGGCAGCGTCAGATGTGTATAAGAGACAG-**GAAGGTCACAGCTGTTTGTTAG**	GTCTCGTGGGCTCGGAGATGTGTATAAGAGACAG-**CATATTAACCTCCCCAACCAG**	239	306
Off target site 2	TCGTCGGCAGCGTCAGATGTGTATAAGAGACAG-**GCAATCAGTTGACTTTAAGTAAGGG**	GTCTCGTGGGCTCGGAGATGTGTATAAGAGACAG-**GAGATCCTGAAAACTGGCTAGC**	206	273
Off target site 3	TCGTCGGCAGCGTCAGATGTGTATAAGAGACAG-**GGCAATGGGAGAGGACATTC**	GTCTCGTGGGCTCGGAGATGTGTATAAGAGACAG-**CACAGGCCTGCAGATTAAATC**	236	303
Off target site 4	TCGTCGGCAGCGTCAGATGTGTATAAGAGACAG-**CCTTCGAGACTCTGGCAGAT**	GTCTCGTGGGCTCGGAGATGTGTATAAGAGACAG-**GTTTGTGGTGTGAAACATGGAG**	211	278
Off target site 5	TCGTCGGCAGCGTCAGATGTGTATAAGAGACAG-**GCATGATAGTCAATTCAGGACCC**	GTCTCGTGGGCTCGGAGATGTGTATAAGAGACAG-**TGTTAGGATAAGAGGGCAGG**	178	245
Off target site 6	TCGTCGGCAGCGTCAGATGTGTATAAGAGACAG-**TGGAAGCATGCAGGGAATG**	GTCTCGTGGGCTCGGAGATGTGTATAAGAGACAG-**GAAAACCCTCCCATCATGGAG**	193	260
Off target site 7	TCGTCGGCAGCGTCAGATGTGTATAAGAGACAG-**GTGACATCAAAGAGGGCATG**	GTCTCGTGGGCTCGGAGATGTGTATAAGAGACAG-**GCTAGCCTCTTTTCTGATAGTG**	243	310
Off target site 8	TCGTCGGCAGCGTCAGATGTGTATAAGAGACAG-**CAGCAATTATGTCATCTGGCTTG**	GTCTCGTGGGCTCGGAGATGTGTATAAGAGACAG-**GGTTATAAACGACCACTTGCTTTAG**	212	279
Off target site 9	TCGTCGGCAGCGTCAGATGTGTATAAGAGACAG-**CCTTCGCACAATGACACTATCC**	GTCTCGTGGGCTCGGAGATGTGTATAAGAGACAG-**CTGAAGTCTGTCTGCATCAAG**	222	289
Off target site 10	TCGTCGGCAGCGTCAGATGTGTATAAGAGACAG-**GGCTCAGTGACAATGAACAGAG**	GTCTCGTGGGCTCGGAGATGTGTATAAGAGACAG-**AGATAGAATGGGGTACTTGGAG**	205	272

Table S10: Primary Antibodies

Antibody	Species	Antibody information	Catalogue no.	Dilution	
				ICC	WB
AIPL1	Rabbit	J. van der Spuy lab, AIPL1 C-terminus	-	1 / 250	-
AIPL1	Rabbit	V. Ramamurthy lab	-	1 / 500	1 / 2500
PDE6A	Rabbit	Proteintech	21200-1-AP	1 / 2000	-
PDE6B	Rabbit	Thermo Fisher Scientific	PA1-722	1 / 1500	-
cGMP	Sheep	BioRad	OBT5055	1 / 100	-
Rhodopsin	Mouse	Millipore	4D2	1 / 1000	-
Cone Arrestin	Mouse	7G6 monoclonal AB		1 / 200	-
Cas9	Mouse	Biolegend	7A9	1 / 500	1 / 2500
FLAG	Rabbit	Proteintech	20543-1-AP	1 / 2000	1 / 20000
GFP-AF488 conjugated	Rabbit	Invitrogen	A21311	-	1 / 2000
mCherry	Rabbit	Abcam	AB167453	1 / 500	-
GAPDH	Mouse	Proteintech	60004-1-lg	-	1 / 10000

Table S11: Secondary Antibodies

Secondary Antibodies	Fluorophore	Company	Catalogue no.	Dilution	
				ICC	WB
Donkey anti-mouse IgG	AF488	Thermo Fisher Scientific	A-21202	1 / 1000	1 / 10000
Donkey anti-mouse IgG	AF555	Thermo Fisher Scientific	A-31570	1 / 1000	1 / 10000
Donkey anti-mouse IgG	AF647	Thermo Fisher Scientific	A-31571	-	1 / 10000
Donkey anti-rabbit IgG	AF555	Thermo Fisher Scientific	A-31572	1 / 1000	1 / 10000
Donkey anti-rabbit IgG	AF488	Thermo Fisher Scientific	A-21206	1 / 1000	
Donkey anti-sheep IgG	AF488	Molecular Probes	-	1 / 1000	

Table S12: Stains

Stain	Fluorophore	Company	Catalogue no.	Dilution
Phalloidin	AF488	Invitrogen / Molecular Probes	A12379	1:40 of 200U/ml solution
DAPI	-	Sigma-Aldrich	D9542	1µg/ml final conc.

SUPPLEMENTAL FIGURES

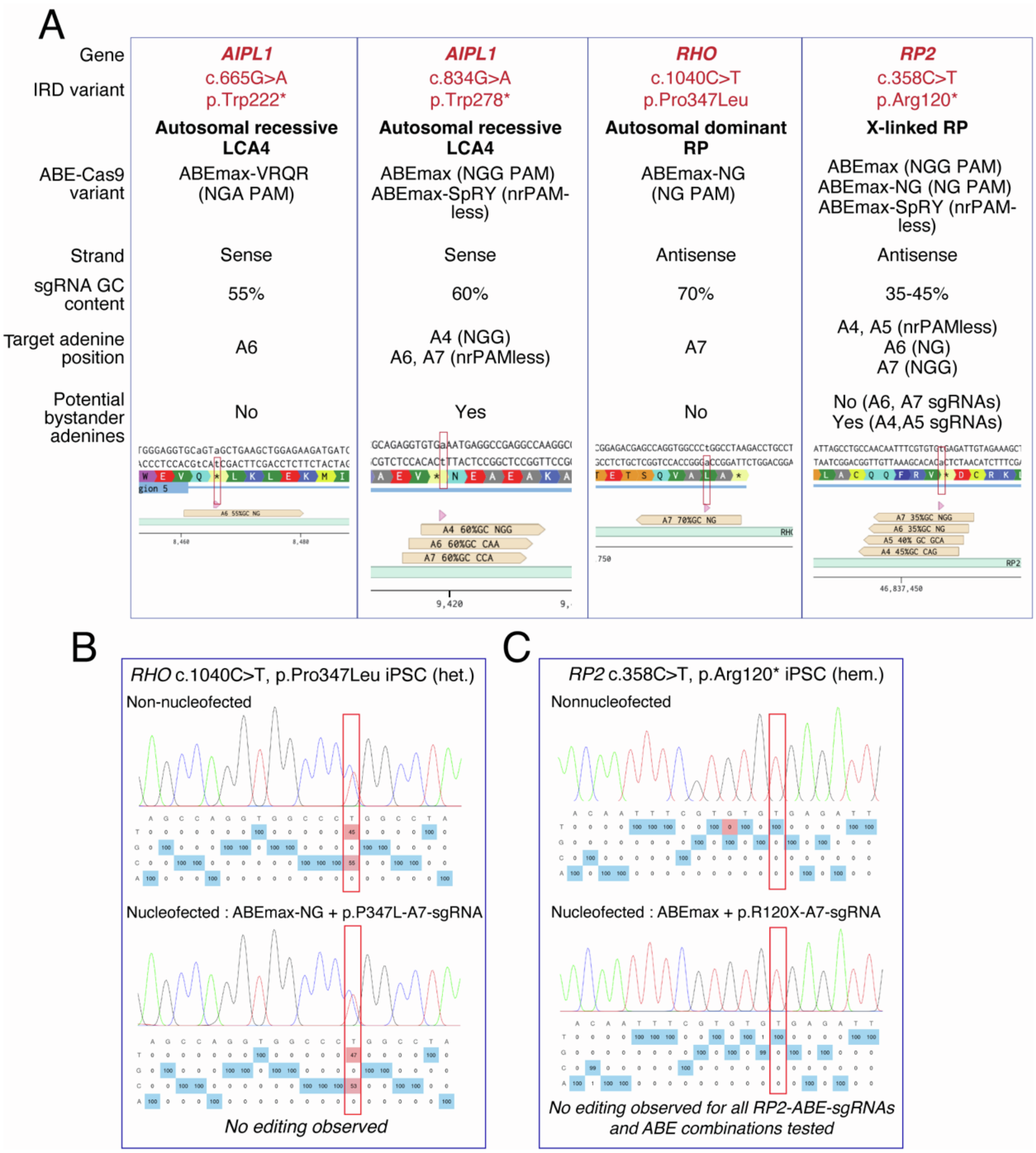

Figure S1: Testing of ABE-sgRNA and base editors in IRD patient iPSCs harbouring variants potentially amenable to correction by adenine base editing, related to Figure 1

(A) Table with details of the four IRD gene variants and characteristics of potential ABE-sgRNA and base editor combinations for each locus.

(B and C) No ABE was observed for either *RHO* c.1040C>T, p.Pro347Leu (B) or *RP2* c.358C>T, p.Arg120* (C) with the ABE and sgRNA combinations tested. Antisense sgRNAs were designed for these loci. The target adenine positions (thymine on the sense strand) are highlighted in the red boxes.

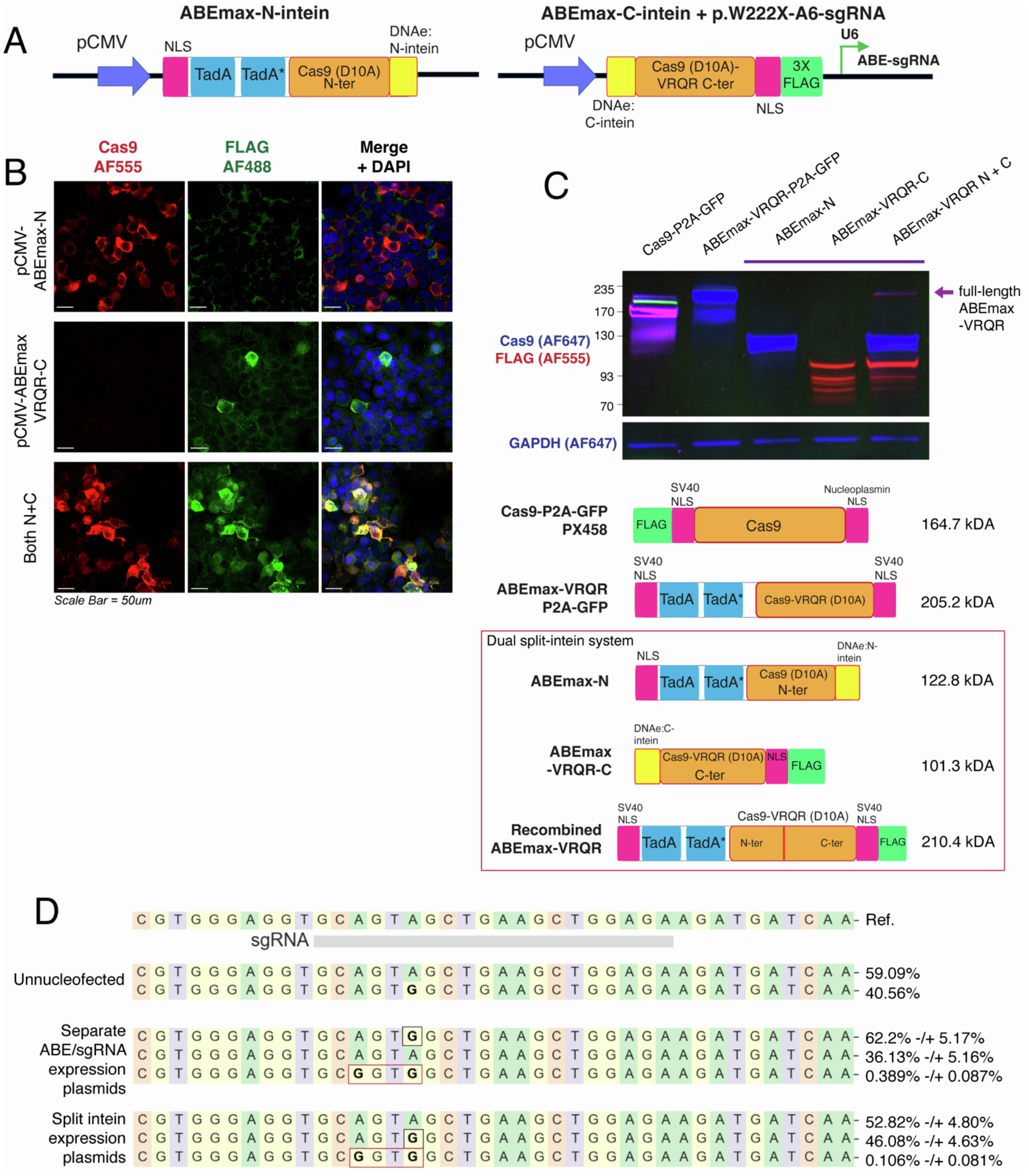

Figure S2: Characterisation of the split intein system for ABEmax-VRQR expression in HEK293T cells, related to Figure 1

(A) Schematic of the dual-plasmid split intein system for expression of ABEmax-VRQR and *AIPL1* c.665G>A-A6-sgRNA.

(B) ICC analysis of transfected HEK293T cells for Cas9 and FLAG expression. HEK293T cells were transfected with expression plasmids for pCMV-ABEmax-N, pCMV-ABEmax-VRQR-C or both plasmids together. The fixed cells were probed with antibodies raised against the N-terminus of Cas9 and FLAG epitopes. As expected, the Cas9 antibody was able to detect ABEmax-N and not the C-terminus portion of the protein, and a positive signal for FLAG was detected in cells transfected with ABEmaxVRQR-C plasmid. Cells transfected with both plasmids showed a high degree of overlap in Cas9 and FLAG positivity.

(C) Western blot analysis of transfected HEK293T cell extracts for base editor protein epitopes. Cas9 and FLAG antibodies (visualised with AF647 (blue) and AF5555 (red) conjugated secondary antibodies respectively) detect the N and C terminus protein halves, with double staining for both at ~210.4kDa (purple band) evident in the N+C lane, indicating that there is recombination of full-length ABEmax-VRQR. Cas9-P2A-GFP and ABEmax-VRQR-P2A-GFP samples were used as controls for antibody labelling and for comparison of band sizes (shown in the schematic). All expression plasmids featured the constitutive pCMV promoter. Endogenous GFP fluorescence was visible in Cas9-P2A-GFP samples. GAPDH (~36kDa) was visualised as a loading control.

(D) HTS analysis of LCA4 iPSC samples nucleofected with separate and split intein expression plasmids. The percentages of amplicons with A and G at position A6 are shifted in samples nucleofected with base editing plasmids. A small degree of bystander editing was observed at position A3. These amplicons were also edited at target A6 (editing at A3 and A6 demarcated by a red rectangle). Average percentages and standard deviations calculated from 3 independent samples.

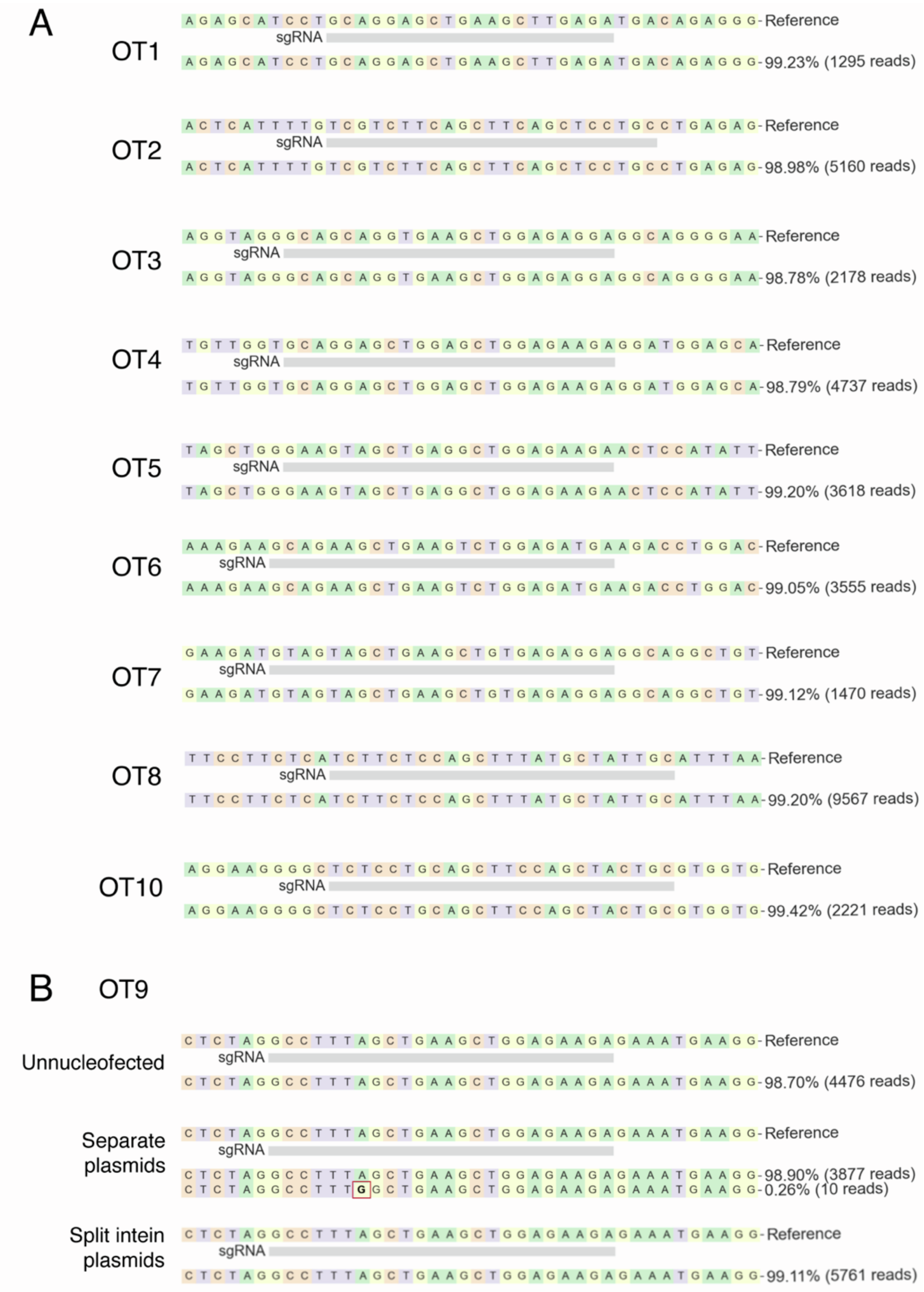

Figure S3: HTS analysis of potential *AIPL1* c.665G>A-A6-sgRNA / ABEmax-VRQR off-target site editing in nucleofected patient iPSCs, related to Figure 1

(A) Off target sites 1-8 and 10 (OT1-8, OT10) showed no signs of off-target editing in iPSCs nucleofected with either separate expression plasmids or split intein expression plasmids (results shown are from analysis of the locus in nucleofected iPSCs).

(B) Low level off-target editing was detected for OT9. This was seen in separate expression plasmid samples and not in split intein samples.

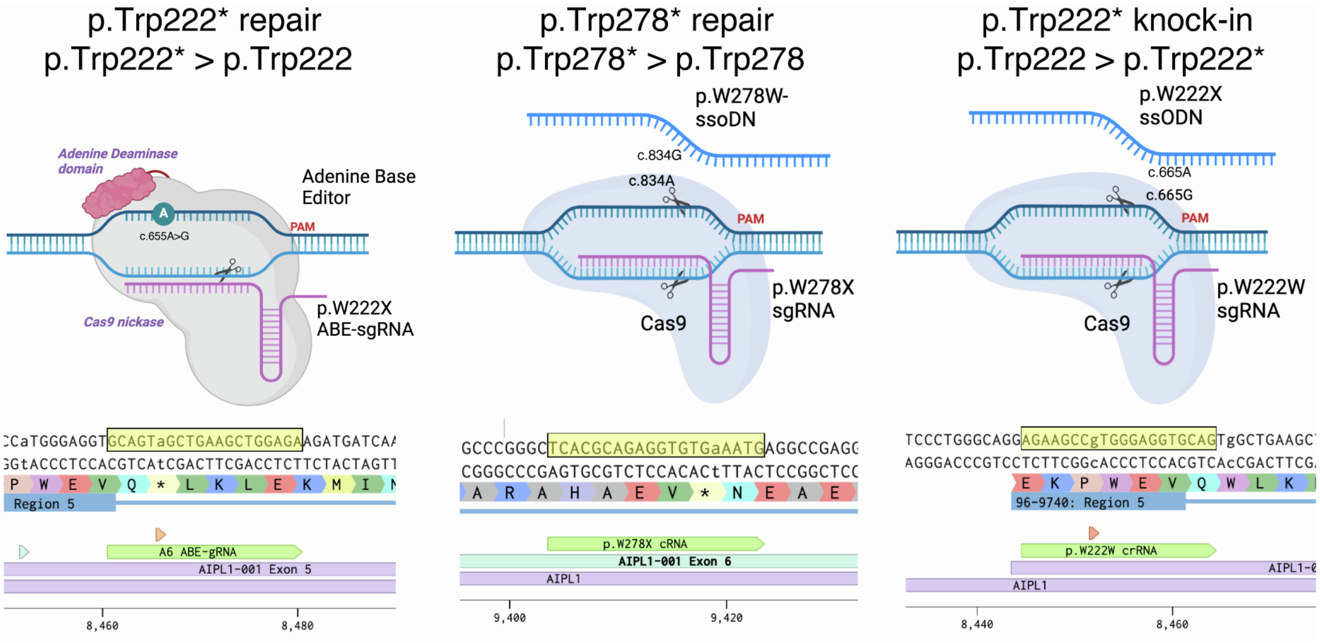

Figure S4: Generation of isogenic lines from compound heterozygous p.Trp222*/p.Trp278* patient iPSCs, related to Figure 2

Schematic diagrams detailing the generation of isogenic iPSC lines. A combination of adenine base editing at AIPL1 p.Trp222* and CRISPR/Cas9-mediated homology-directed repair at AIPL1 p.Trp278* and at p.Trp222 were used to generate two different heterozygous repair lines (Trp222* repair and Trp278* repair respectively) and a double repair line (both Trp222* and Trp278* repair), in addition to a new LCA4 model that is homozygous for p.Trp222*. Sequences for crRNAs and ssODNs are in **Table S4.**

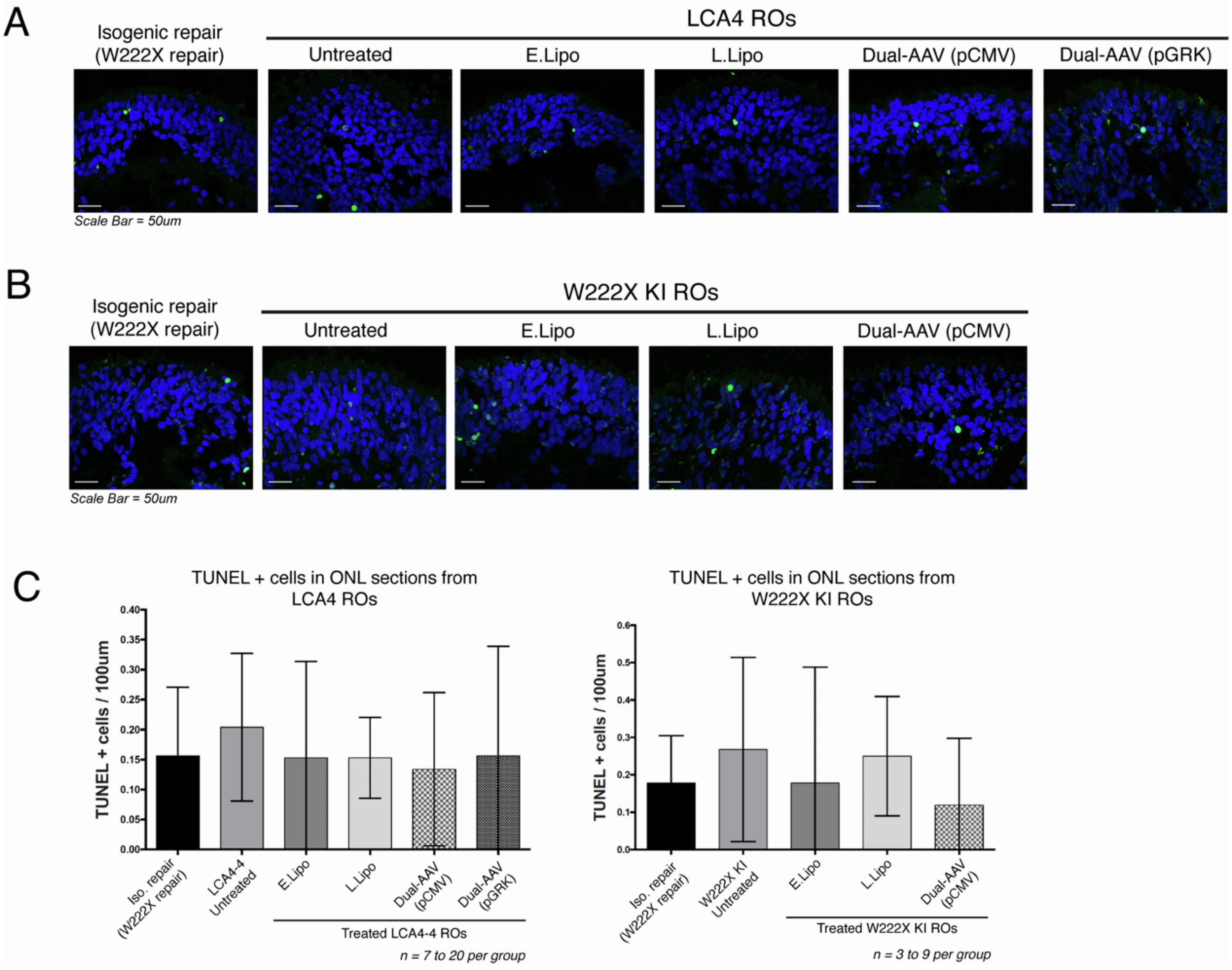

Figure S5: Analysis of apoptosis in isogenic repair and LCA4 model (untreated and treated) retinal organoids, related to Figure 3

(A) and (B) Representative images of the ONL region in RO sections from (A) W31/D217 LCA4 ROs (untreated and treated), and (B) D182 Trp222* KI ROs (untreated and treated), stained for TUNEL positivity (green). DAPI in blue. TUNEL positivity was not visually different to age-matched isogenic repair (Trp222* repair) ROs. Scale bars = 50µm.

(C) Comparison of the number of TUNEL positive cells/100µm ONL width in RO sections from LCA4 and Trp222* KI ROs. There is no significant difference between any of the groups (n = 7-20 per group). Two-tailed parametric t-test, Welch’s correction.
